# Study protocol: Neuro-inflammatory parameters as mediators of the relationship between social anxiety and itch intensity: A cross-sectional, controlled laboratory study in patients with psoriasis and healthy skin controls

**DOI:** 10.1371/journal.pone.0281989

**Published:** 2023-03-16

**Authors:** Marcel Schepko, Katharina C. Stumpf, Susanne Tumala, Eva M. Peters, Jörg P. Kupfer, Christina Schut

**Affiliations:** 1 Institute of Medical Psychology, Justus-Liebig-University Giessen, Giessen, Germany; 2 Department of Psychosomatic Medicine and Psychotherapy, Psychoneuroimmunology Laboratory, Justus-Liebig-University Giessen, Giessen, Germany; Kansai Medical University: Kansai Ika Daigaku, Institute of Biomedical Science, JAPAN

## Abstract

**Introduction:**

Psoriasis (PSO) is a disease that in the majority of patients is accompanied by itch, which imposes a great burden and positively relates to anxiety. *Social* anxiety, a facet of anxiety associated with social withdrawal, may be a predictor of itch intensity in this patient group. Moreover, anxiety is linked to the secretion of neuroendocrine and inflammatory parameters such as substance P (SP), interleukin (IL)-6 and IL-17, which are also related to itch. In this research project, we investigate first, whether there is a direct relationship between social anxiety and itch intensity in patients with PSO and second whether the secretion of SP, IL-6 and IL-17 in the skin mediates this relationship. Additionally, PSO-patients are compared to healthy skin controls regarding their level of social anxiety, itch intensity and the secretion of SP, IL-6 and IL-17.

**Methods and analyses:**

For study 1, we aim to recruit 250 psoriasis patients and 250 healthy skin controls who complete questionnaires to assess social anxiety, itch intensity and control variables (e.g. sociodemographic variables and severity of PSO). A linear hierarchic regression will be used to determine whether social anxiety significantly contributes to itch intensity. In study 2, we plan to apply the suction blister method to 128 patients and healthy skin controls recruited from study 1 to determine SP, IL-6 and IL-17 in tissue fluid extracted from the skin. A mediation analysis will be conducted using the SPSS-macro PROCESS to test whether the relationship between social anxiety and itch is mediated by SP, IL-6 and IL-17.

**Trial registration numbers:**

DRKS00023621 (study 1) and DRKS00023622 (study 2).

## Introduction

### Psoriasis (PSO) and itch, a self-reinforcing misalliance

PSO is a chronic inflammatory skin disease characterized by a hyperactivation of T-helper cell (TH) type 1 and 17 immune responses and related hyperproliferation of keratinocytes leading to visible skin lesions, especially at the elbows, scalp, lower legs and knees [1–4]. PSO affects about 2.5% of the German adult population and in the majority of patients it is accompanied by itch [5–9]. In fact, itch—in many cases—leading to scratching behavior [10] is regarded as the most bothersome symptom by PSO-patients [11]. This further impedes the patients’ appearance and in the following may lead to feelings of stigmatization, *social* anxiety and isolation, especially due to the stares of other people and negative self-assessment [12–14]. Closing a vicious circle, levels of internalized stigmatization have been shown to be related to PSO-severity and the visibility of lesional skin [14]. Overall, PSO often so dramatically impedes the well-being of the patients that PSO-patients show increased levels of depression and suicidal ideation compared to healthy controls [15–18].

### Itch links with psychological factors

Itch is related to a number of psychological factors including stress, personality traits, depression and self-consciousness [19–23]. In a mice model, it has been shown that social deprivation increased both, anxiety related behavior and self-scratching responses [24]. Other work demonstrates that acute [25] and chronic itch [26, 27] play an important role in triggering behavior, which reflects states of anxiety in mice. This effect seems to be associated with mast cell activation as mice that received injections of compound 48/80 after 10 days of water avoidance stress, showed increased scratching responses compared to a control group without stress induction [28]. Corresponding to these observations in mice, patients with dermatologic conditions who reported increased anxiety, also reported more severe itch, which in turn was aggravated by scratching behavior [29]. While scratching as response to induced itch was judged as relieving in skin healthy participants [30], PSO-patients perceive self-scratching due to disease related itch as burdening in various daily social situations [31], and show a positive relationship between anxiety and itch [32–34]. In turn, selective serotonin reuptake inhibitors reduce itch in dermatologic and psychosomatic patients [35], and giving escitalopram significantly diminishes anxiety levels and itch in PSO-patients with psychiatric disorders [36]. The above mentioned studies demonstrate the important role of the link between itch and anxiety in PSO-patients. Social anxiety represents a facet of anxiety that deserves special interest in this context. It is related to more severe psychological comorbidities such as depression and suicidality, which also play a role in psoriasis [15, 37–40]. Even though it has been shown that itch is linked to anxiety in patients with PSO and atopic dermatitis (AD) [11, 32, 41] the role of social anxiety has not been investigated in detail in PSO-patients.

### Neuroendocrine and inflammatory parameters relate to itch and anxiety

There are a number of neuroendocrine and inflammatory parameters interacting with each other, which are related both to the intensity of itch as well as to anxiety. Of these parameters, we consider substance P (SP), IL-6 and IL-17 of special interest. Concerning itch, injected SP was shown to induce it in mice [42, 43], in healthy human controls [44], as well as in PSO-patients [45]. Within this context, it was recently shown that in PSO-patients and in patients with atopic dermatitis, the amount of SP and its neurokinin-1 receptor (NK-1R) receptor was significantly increased in nerve fibers in itchy compared to non-itchy skin of the same patients and to healthy controls [46]. In the same study, gene fold changes of IL-6 and IL-17A were overexpressed in itchy skin of PSO-patients. Of note, SP is capable of liberating IL-6 and IL-17 [47, 48], and IL-17 has also been shown to further release IL-6 [49]. Moreover, higher levels of IL-6 have been found in itchy skin of patients with different diseases. With this respect, it was found that IL-6 concentration were significantly related to scratching behavior after intradermal injections of calcium phosphate in mice. In the same study, in human subjects with chronic kidney disease, IL-6 concentrations were significantly increased in itchy compared to non-itchy skin [50]. In addition, an increased amount of nerve fibers containing IL-6 was found in lesional compared to non-lesional skin of patients with the itchy skin diseases atopic dermatitis and prurigo nodularis [51]. More recently, this finding was supported by a positive relationship between pruritus severity and IL-6 in patients with prurigo nodularis [52]. In addition, IL-17-antagonists were shown to reduce itch in PSO-patients [53–55].

These neuroendocrine and inflammatory parameters are also associated with anxiety [56–61]. First of all, the availability of the NK-1 receptor in the amygdala was shown to be significantly associated with anxiety-like personality traits in healthy individuals [62]. Also, the induction of itch by intradermal injections of histamine, which can be liberated by SP [44], caused anxious behavior in mice [25]. Moreover, IL-6 has been shown to be significantly elevated in patients with clinically diagnosed anxiety disorder compared to non-anxious individuals [56]. Similarly, IL-17 concentrations were shown to be elevated in mononuclear blood cells of patients with generalized anxiety disorders compared to healthy controls [63]. In both groups, IL-17 levels significantly increased after SP was added to the cells in a concentration that mimicked a stressful event, which supports the biological link between anxiety, SP and inflammation.

Due to the known relationships between these parameters and anxiety on the one hand and itch on the other hand, the link between anxiety and PSO-itch could be mediated by SP, IL-6 and IL-17 [64]. In this study, we investigate whether social anxiety can predict itch and whether this relationship is mediated by the release of these neuroendocrine and inflammatory parameters. To study this interaction on the local stress mediator release level, we here plan to use a minimally invasive skin sampling technique, the suction blister method [65], which allows the determination of these neuroendocrine and inflammatory parameters directly in the epidermis and interstitial cutaneous fluid.

### Objectives

**Fig 1:**
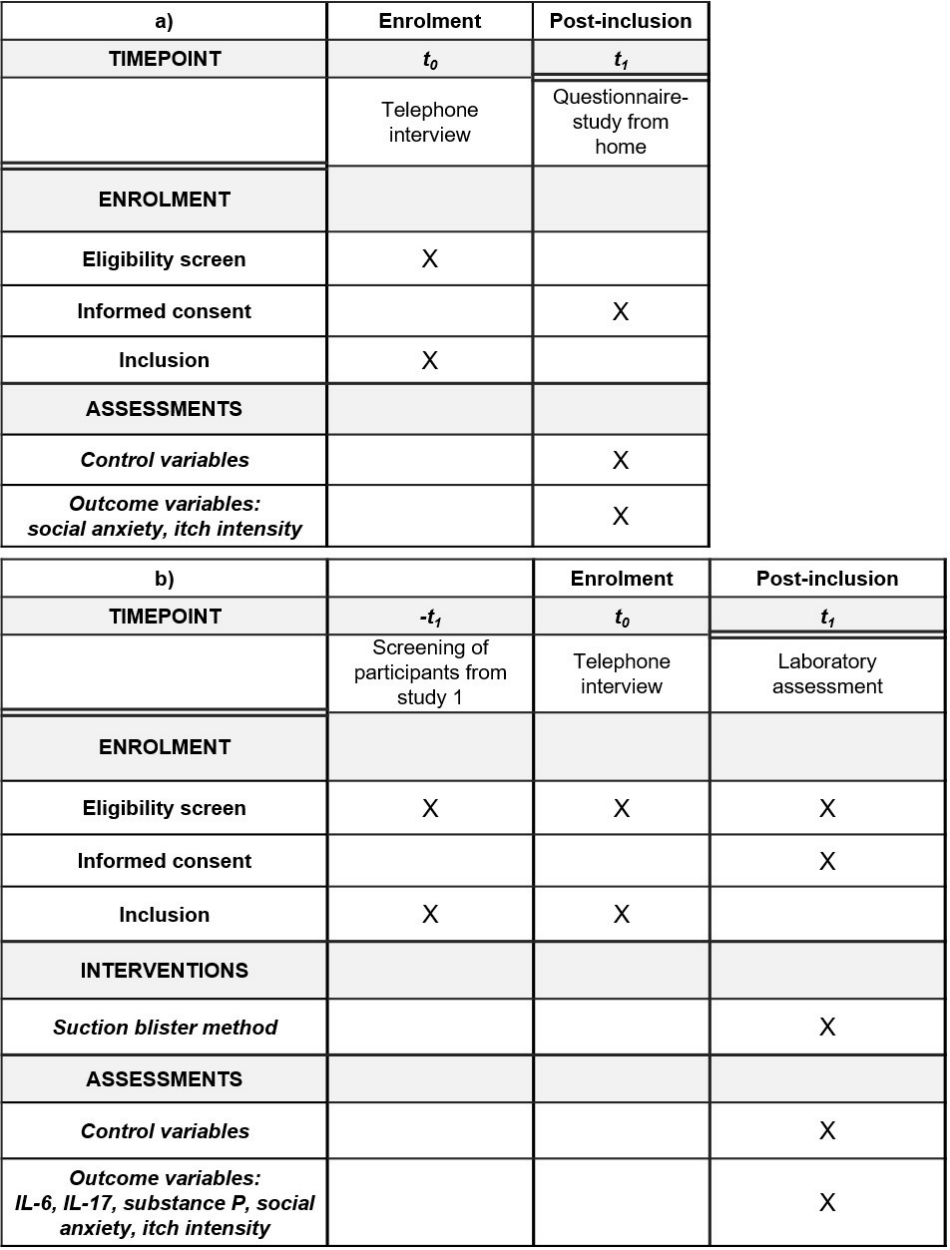
Spirit schedule. Schedule of enrolment and assessments of study 1 (Fig 1a) and study 2 (Fig 1b).

The first aim of the research project is to investigate whether social anxiety is a predictor of itch in PSO-patients (study 1). The second aim of this research project is to answer the question whether IL-6, IL-17 and SP mediate the relationship between social anxiety and itch intensity in PSO-patients. In addition, we investigate, whether healthy skin controls and PSO-patients differ regarding social anxiety and/ or the release of SP, IL-6 and IL-17 (study 2; also see spirit schedule, Fig 1)

**Fig 2:**
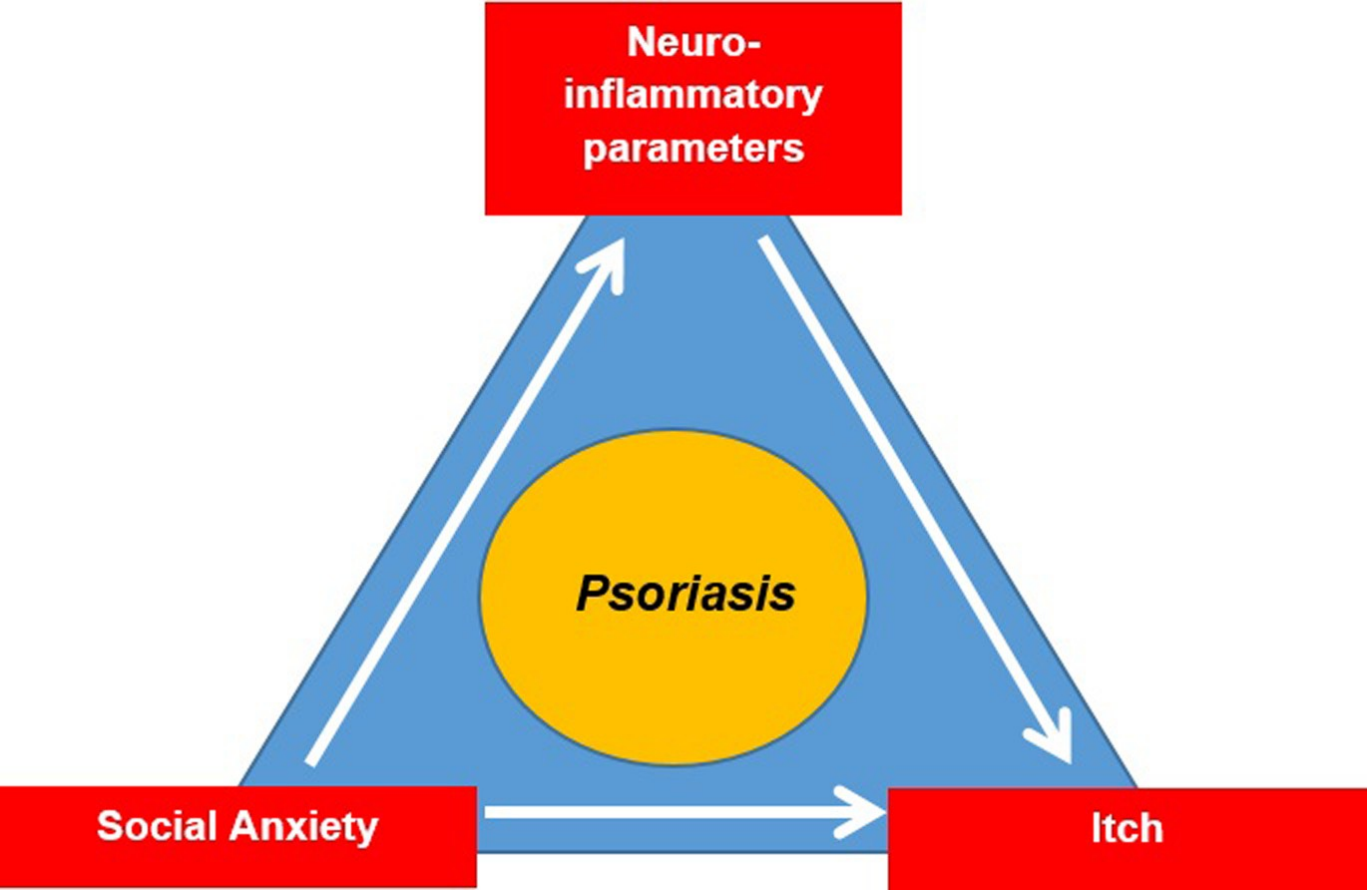
The aim of study 1 is to investigate the relationship between social anxiety and itch intensity. The aim of study 2 is to explore the mediating effect of neuroendocrine and inflammatory parameters in the relationship between social anxiety and itch in PS-patients.

For an illustration of the research questions of this research project, see Fig 2.

## Materials and methods

### Study design, patient recruitment, inclusion and exclusion criteria

Study 1 is a cross-sectional observational questionnaire study to answer the first research question (whether itch and social anxiety are related in PSO-patients) and to recruit PSO-patients willing to perform suction blister sampling for study 2. Study 2 is a controlled laboratory study, in which we plan to include a selected subpopulation of high and low socially anxious PSO-patients and healthy skin controls as described below. In order to recruit participants for study 1, PSO-patients will be informed about the study by written and oral announcements, information by their dermatologists as well as via websites of German psoriasis networks. Healthy skin controls will be recruited via oral and written announcements. In case patients/healthy skin controls are interested in participation in study 1, they will be contacted by staff from the Institute of Medical Psychology of the Justus-Liebig-University of Giessen via telephone, and receive further information on the study. During this telephone call, inclusion and exclusion criteria will be checked. In order to be included in study 1, participants need to be between 18–65 years old, know the German language well enough to be able to fill in the questionnaires adequately, and in case of PSO-patients have been clinically diagnosed with PSO at least six months prior to inclusion. PSO-patients and controls with other itchy (skin) conditions such as bullous disorders are excluded. Also, individuals who currently receive or have received psychotherapeutic/ psychopharmacological treatment due to a psychological/ psychiatric disorder during the last six months are excluded. Further exclusion criteria for study 2 are a diagnosis of any transmissible disease, and treatment with biologicals, because these can interfere with neuroendocrine and immune parameter measurements [66]. In general, we strictly protocol medical and psychological conditions before inclusion and at the beginning of the examination in order to be able to assess how comorbidities affect our findings.

### Study procedure

The research project is divided into two studies. Study 1: After inclusion, validated self-report instruments and a consent form will be sent to the participants by mail, which they are to return to the Institute of Medical Psychology in pre-stamped envelopes provided with the package. The questionnaire sets include items to assess sociodemographic information, social anxiety and itch intensity. In case of PSO-patients participants will additionally report about the severity of their PSO. The data will be transferred to IBM® SPSS® and stored pseudonymously in line with the data protection regulations. For participation in study 1, participants are financially reimbursed. They are also asked to leave their contact details, if they are interested in participation in study 2.

Study 2: Patients and healthy skin controls will be selected from the pool of study 1, who showed interest to participate in study 2. They will be informed about the procedures involved in study 2 in a telephone conversation. In this phone call, the inclusion and exclusion criteria (see above) will be assessed. After inclusion, participants will be invited to the laboratory of the Institute of Medical Psychology. Participants will be habituated to the suction blister method by verbal instructions of the experimenter, and a demonstration of the loudness and handling of the suction pump (session 1; habituation). Participants are informed that they can withdraw from the study at any time without giving reasons. Afterwards, they give their informed consent and have a standardized lunch break before the actual induction of blisters on the skin (session 2). At the beginning of the second session in the laboratory, participants will be asked to fill in the same questionnaires as in study 1. In addition, in study 2, two further questionnaires will be deployed to assess the current psychological health, and current/ general anxiety and depression (see below). Afterwards, the suction blister pump will be used to induce blisters. This allows the collection of blister fluid and the roof of the blisters for determination of neuroendocrine and inflammatory parameters therein. In total, both sessions of study 2 will last 5.5 hours approximately. All participants will receive a financial compensation for participation in study 2. Details and unforeseen events during the trial will be protocolled by the experimenter.

### Psychometric measures

#### 1) Social anxiety

Social anxiety is measured in both studies by means of the *social anxiety disorder scales* (SOZAS) [67]. These include the *social interaction anxiety scale* (SIAS) and the *social phobia scale* (SPS). Both are validated German questionnaires, which contain 20 items each that need to be answered on a 5-point scale. The SIAS measures interactional anxiety, the SPS measures anxiety in evaluation situations. The items in both questionnaires do not refer to a specific time period. In this research project, subjects will be categorized as being either high (HSA) or low (LSA) socially anxious according to a median split of the SIAS scores in order to determine whether these groups differ in itch intensity and the secretion of assessed neuroendocrine and inflammatory parameters.

#### 2) Itch intensity

In studies 1 and 2, average and maximum itch intensity (base line itch) during the last 24 hours will be assessed by means of a visual analogue scale (VAS 0–10) with the two poles "no itching" (0) and "worst imaginable itching" (10). PSO-patients and healthy skin controls will also be asked to answer an item on the occurrence of chronic itch. In case of PSO-patients, the current itch-intensity in relation to the worst itch-intensity experienced due to PSO will also be assessed. Specifically, participants are asked to indicate whether the current itch is as severe, minimally better, somewhat better, significantly better or much better than the worst itch they have ever experienced due to PSO.

As the application of the suction blister method might induce itch [68–70] and mechanical skin stretching has been shown to increase substance P in the skin of mice [71], we consider maximum induced itch due to the application of the suction blister method as control variable. Itch will therefore be measured by means of a VAS (0–10) every 30 minutes (up to six times) throughout suction blister induction.

### Biologic measures

**Fig 3:**
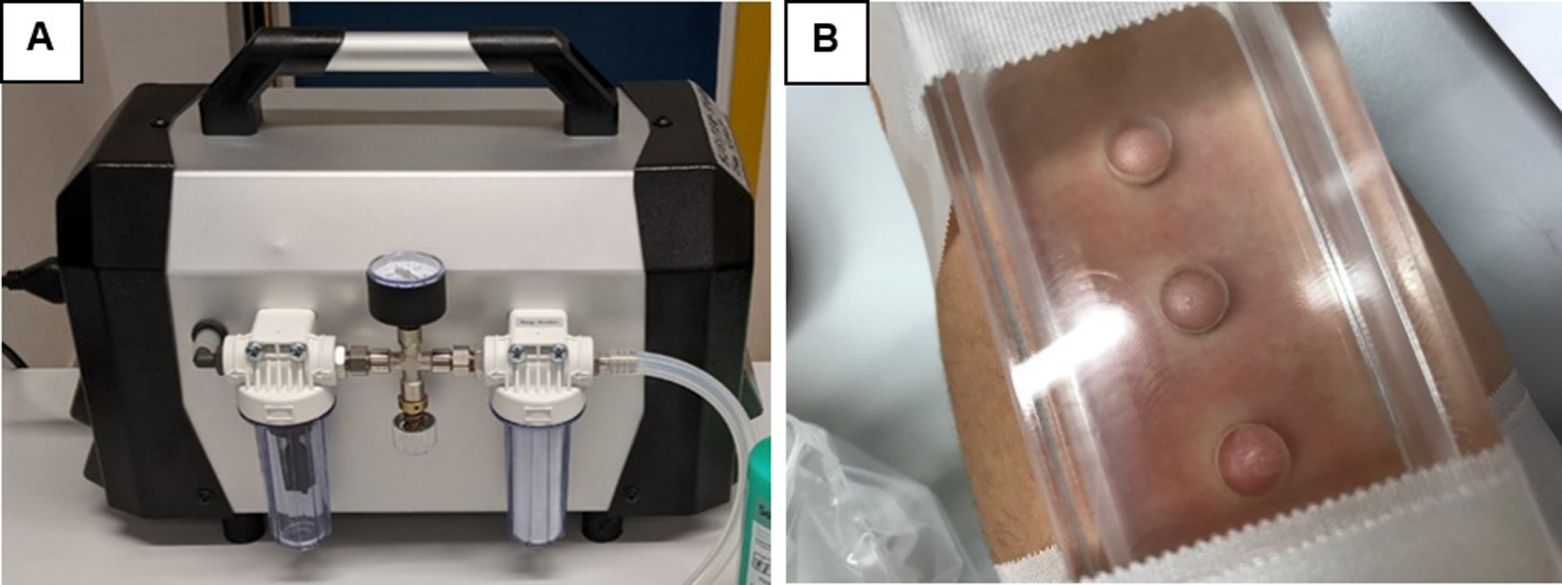
Illustration of the suction blister methods. A) Suction blister pump (Metzger Technik®, Vaihingen an der Enz: Delta-Typ Labovac, vaccum maximum ≈ -870 mbar) B) three skin blisters containing tissue fluid induced by the use of the suction blister method after three hours.

Material for biological assessments will be obtained by use of the suction blister method. This method was developed more than 50 years ago [65] and allows minimal-invasive ablation of the uppermost layers of the epidermis from the basement membrane separating the epidermis from the dermis. Thereby, blisters form above the basement membrane without injury of the underlying structures during application of a constant negative pressure of 200–300 mmHG. This negative pressure will be applied and controlled by a finely adjustable suction pump (Metzger Technik®, Vaihingen an der Enz: Delta-Typ Labovac, vaccum maximum ≈ -870 mbar), and a plexiglas suction chamber constructed at a workshop of the department of veterinary medicine at the Justus-Liebig-University of Giessen according to the design by Eva Peters based on previous experiences with the method and published methodological protocols [72–74]. It has 3 circular, 8 mm diameter openings as shown in Fig 3, which allow induction of 3 blisters at the same time by applying a negative pressure. In case of PSO-patients, the blisters are induced in such a way that they are located at least 3 cm from the nearest lesional skin area on the supinated forearm of the non-dominant hand side. This process results in the transfer of interstitial fluid and cells from the epidermis and dermis into the blister cavity, including intercellular proteins and other messengers such as neurotransmitters, neuropeptides and cytokines as well as cells of the immune system [75–78].

Blister formation is usually completed after 1–3 hours and the individual duration will be documented. After completion of suction blister induction, the fluid underneath the blister roof is aspirated under sterile conditions from the blister cavity by using an insulin syringe, transferred to an Eppendorf tube (Sarstedt, Nümbrecht: safe seal tube, volume = 1,5ml) and centrifuged to separate cells contained in the fluid from the liquid phase. The supernatant is then stored at -80°C until analysis of the neuroendocrine and immune parameters is performed. For future analyses of mRNA expression, epigenetic methylation patterns or flow cytometry, the cells obtained by centrifugation and the blister roof areal are also collected and stored at -80°C. It is not planned to provoke an immune response prior to suction blister induction as done in other studies [79]. Instead, native material will be collected and stored to examine the in vivo disease conditions and their link with specific social anxiety conditions.

#### 1) Neuroendocrine parameters

Levels of SP will be determined in the blister fluid obtained by use of the suction blister method as described above. Herefore, we will apply ELISA assays (IBL International, Hamburg, Germany) according to modified manufacturer’s recommendations.

#### 2) Inflammatory parameters

IL-6 and IL-17 will be determined by ELISA assay as described above and additional comprehensive panel of mediators of innate and learned immunity might be determined in future studies using Cytometric Bead Array Analysis (Bender MedSystems, eBioscience, Frankfurt, Germany).

### Control variables

In both studies, person-related variables (age, gender, BMI, smoking, use of oral contraceptives, and social status) will be assessed as control variables to control for standard confounding factors. The self-administered patient assessed severity index (SA-PASI) will be used in PSO-patients to measure the severity of PSO during the last 24 hours before examination [80]. In addition, the current psychological health status will be assessed in study 2 with the short form of the German Health Questionnaire for Patients (PHQ-D) [81], and general and current anxiety and depression will be assed with the State-Trait-Anxiety-Depression-Inventory (STADI) [82]. In addition, the suction-blister-pump will be applied during the time from 12 to maximally 3.30pm in order to consider the alteration of neuroendocrine and inflammatory parameters during the day [83–85].

### Statistical analyses

Statistical analyses will be performed using SPSS and the macro PROCESS for SPSS [86–88]. In order to answer research question of study 1, Spearman’s rank-order correlation analyses will be conducted first in order to see which of the control variables are significantly related to itch intensity in PSO patients. These will then be included in step 1 of the subsequent linear hierarchical regression model. Social anxiety measured by the SIAS and SPS will be considered as predictor variable in step 2 of the analyses. Maximum and average itch intensities represent the criterion variables

To answer the research question of study 2, mediation analyses will be conducted using the SPSS macro PROCESS [86–88]. In these analyses, control variables significantly associated with itch intensity as well as social anxiety will be included as possible predictor variables, neuroendocrine and inflammatory parameters as mediators, and average as well as maximum itch intensity as criterion variables. If we find significant correlations between maximum induced itch, and the neuro-inflammatory parameters, we will exploratively conduct a regression analysis with the maximum induced itch as end-point.

Separate analyses will be conducted for patients and controls. To determine the required sample size, we performed an analysis using G*Power [89] to extrapolate the sample size for study 2. Based on correlational studies regarding itch and anxiety [33] as well as on neuroinflammatory parameters and itch [52] in PSO-patients we expect small to medium sized effects (f^2^ = .10). The estimation with an α of 0.05 and a β of 20 revealed that 64 healthy skin controls and 64 PSO-patients are to be included in study 2. Small to moderate effects can be assumed because, on the one hand, only extreme groups are examined and, on the other hand, small to moderate correlations between anxiety and itch intensity (*r* = .179; *p* = .003) have been found in PSO-patients [33] and between neuroinflammatory parameters and itch intensity (*r* = .60; *p* < .01) [52]. In case, that patient recruitment for the studies is slower than anticipated, we would aim to gather additional personnel and material resources to improve the recruitment rate over time. Since study 2 has more strict inclusion and exclusion criteria on e.g. medication and comorbidities than study 1 (see inclusion and exclusion criteria), we expected a reduction in participants who match these premises. In addition, motivational factors are to be considered, as in contrast to study 1, study 2 will be conducted in a laboratory setting. We therefore estimated that n = 500 participants need to be included in study 1 to achieve the calculated sample size of n = 128 participants for study 2. As there is no cut-off value in the user manual of the SIAS [90] to categorize somebody as low socially anxious, we planned to use percent range ≤ 25 in order to categorize 32 participants per group as being low socially anxious. Due to the pandemic situation, participant recruitment during the course of 2020 and 2021 was and still is very difficult. Therefore, we had to change this inclusion criterion for study 2 and now invite all participants of study 1 irrespective of their social anxiety scores to take part in study 2. High and low socially anxious participants will now be identified by median split. Statistical analyses will be conducted when the targeted sample size (study 1: *N* = 500, study 2: *N* = 128) has been achieved or further inclusion of participants is no longer possible due to the end of funding. No intermediate data analysis is planned. Data collection started after ethic approval in December 2020 for study 1 (number of currently assessed patients n = 234; number of currently assessed healthy controls n = 174), and in September 2021 for study 2 (number of currently assessed patients n = 27; number of currently assessed healthy skin controls n = 15). Data collection will be continued until the planed sample sizes are reached or as long as funding is available.

### Ethics

The study was approved by the Institutional Review Board at the Department of Medicine at the Justus-Liebig-University of Giessen and by the State Medical Association of Hessia. Participants take part in this study voluntarily. They are informed about the study design and possible risks of the application of the suction blister method in detail and give their written consent before participation.

### Patient, public involvement and dissemination

Patients were not involved in planning of the study. However, members of the university, involving staff from multiple medical disciplines discussed the research project, and made suggestions how to improve the study design before a decision was made regarding funding of the study. These suggestions led to the inclusion of a healthy skin control group. Trial results will be disseminated by publication in scientific journals and by conference contributions.

## Discussion

To the best of our knowledge, the current research project is the first to investigate the role of *social* anxiety in PSO-patients in a very detailed manner. It will investigate if neuroendocrine and inflammatory parameters mediate the relationship between social anxiety and itch in these patients. This will shed light on the question if mental health issues contribute to disease burden in PSO-patients and whether this involves biomolecular processes that can worsen inflammation. State of the art validated methods and instruments will be used to answer these questions by combining self-report and minimally invasive biological sampling [65, 67, 72–74, 80–82]. The advantage of the employed biological sampling method, the suction blister method, is that it allows the determination of the inflammation and neuropeptidergic parameters IL-6, IL-17, and SP extracted directly from the skin. Most previous studies on neuro-immune interaction in skin were conducted in mice due to the sampling possibilities in this setting. Hence, the relationship between psychological factors, neuro-immune activation and symptoms of psoriasis can be studied in a human population in the here proposed setting.

Limiting factors of the current research project are that the biosampling procedure itself might possibly cause arousal. To rule out this possibility, participants will be informed about the suction blister method thoroughly in order to habituate to the application of this method. Therefore, we expect that neuroendocrine and inflammatory parameters can be determined in relation to their overall social anxiety condition. Due to the cross-sectional study design, conclusions regarding social anxiety as *cause* of itch cannot be drawn from the data [91]. Also, itch will be measured by self-report only and not by video-recording of scratching behavior. In general, participants must fulfill several inclusion criteria, which limits the external validity [92]. A typical PSO-patient might come with a variety of comorbidities that could conflict with a study design allowing a high internal validity. Overall, we are aiming for a moderate internal and external validity. Thus, we exclude participants who take certain medications (e.g. SSRIs, see Methods), but include individuals with highly prevalent conditions in PSO-patients, such as the metabolic syndrome [93]. In the current research project, we investigate whether social anxiety is a significant predictor of itch mediated by neuroendocrine and inflammatory parameters. Of no less importance may be the question whether itch leads to increased social anxiety. Thus, future prospective studies could e.g. investigate whether the relief of itch diminishes social anxiety mediated by visible lesions of the skin, such as marks or scars. The importance of consequences of scratching has already been shown in such a way that bleeding was the most relevant aspect linked to stigmatization in PSO-patients [94].

## Supporting information

S1 Checklist SPIRIT 2013 checklist: Recommended items to address in a clinical trial protocol and related documents*. (PDF)

S1 File (PDF)

S2 File (DOCX)
