# Supplementary material for: Study protocol: Neuro-inflammatory parameters as mediators of the relationship between social anxiety and itch intensity: A cross-sectional, controlled laboratory study in patients with psoriasis and healthy skin controls
Source: PLoS One. 2023 Mar 16;18(3):e0281989. doi: 10.1371/journal.pone.0281989 (PMC10019658; doi:10.1371/journal.pone.0281989)
Supplement: S1 File — (PDF) [file pone.0281989.s002.pdf]

Translated with www.DeepL.com/Translator (free version), but modified as the translation was not suitable at every site.

Ethics Application Attachment D:
Accurate clinical study protocol with detailed biometrics
Title: Neuro-inflammatory parameters as mediators of the association between social anxiety and itch intensity in psoriasis patients.

Aim and motivation of the study

Psoriasis is a chronic inflammatory skin disease that is associated with itching on the one hand and feelings of stigmatization on the other hand in the majority of patients. Stigmatization leads to social anxiety with avoidance behavior and social withdrawal. A possible pathogenetic relationship between social anxiety and itch can be inferred, as psoriasis patients report elevated anxiety levels and anxiety in psoriasis patients correlates significantly positively with itch intensity. However, this relationship between social anxiety and itch has not been investigated to date. Social anxiety in particular may lead to the initiation of a physiological response to social stress situations such as the release of neuroinflammatory parameters in excess and increase itch. IL-6, IL-17, and substance-P are parameters involved in increased anxiety and itch and will therefore be investigated as mediators of the relationship between social anxiety and itch in this research project (Fig. 1). The aim of the first study is to determine the direct relationship between social anxiety and itch intensity in psoriasis patients. In a second study, neuroinflammatory parameters will be determined in selected patients from study 1 using the minimally invasive suction blister technique to investigate whether these represent significant mediators in the association between social anxiety and itch.

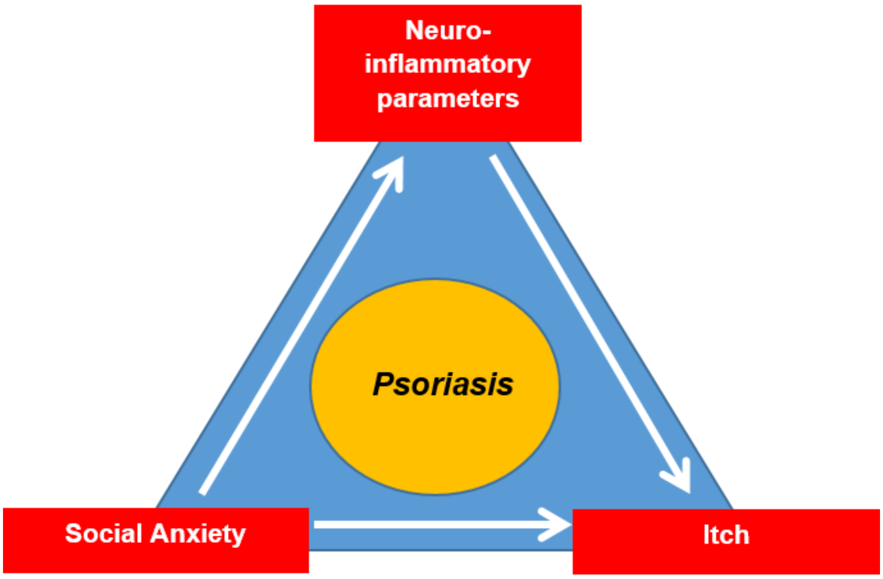

Figure 1. The aim of the study is to investigate the mediating effect of neuroinflammatory parameters in the association between social anxiety and itch in psoriasis.

State of research

In the chronic inflammatory skin disease psoriasis, which affects about 2.5% of the German population, itching is a common symptom with a prevalence of 60-90%, which is perceived as extremely unpleasant and distressing (Globe et al., 2009; Augustin et al., 2010; Szepietowksi & Reich, 2016; Schut et al., 2019). Psoriasis patients report stigmatization (Alpsoy et al., 2017; Sommer et al., 2019), which can be explained by the altered appearance of the skin and scratch lesions. Several studies show a positive association between itch intensity and anxiety in this patient group (e.g., Mrowietz et al. 2015; Zachariae et al., 2012). Animal models also show that more spontaneous scratching behavior occurred in mice in which unpredictable stress was applied for 28 days than in the control group (Wang et al., 2018). In addition, it has been observed that acute (Sanders et al., 2019) and chronic itch (Zhao et al., 2018; Wang et al., 2019) in mice leads to behaviors indicative of anxious symptomatology.

An itch-anxiety cycle is assumed (Sanders & Akiyama, 2018), which must be broken in patients with chronic itch. In this circle, special attention must be paid to social anxiety as a facet of anxiety that leads to avoidance behaviors and social withdrawal. These can thus entail other typical psychological problems such as depression and suicidality, which are observed more frequently in psoriasis patients (Liang et al., 2019). The very important connection between social anxiety and itching in psoriasis patients has not been recorded so far and will therefore be part of the research project (research question 1).

Current research suggests that IL-6, IL-17, and substance P play a role in itch development in general and the latter in psoriasis in particular (e.g., Konda et al., 2015; Théréné et al., 2018; Zeidler et al., 2019; Nattkemper et al., 2018). On the other hand, these neuroinflammatory parameters are also associated with anxiety (e.g., Muñoz & Coveñas, 2014; Liu et al., 2012; Leff Gelman et al., 2019). Accordingly, the link between anxiety and psoriasis symptoms could be mediated via the release of neuroinflammatory cytokines (Connor et al., 2015). However, this has not yet been investigated and is therefore the focus of the planned project (question 2).

As a secondary question, we will investigate whether socially low and highly anxious skin-healthy controls differ in their local release of neuroinflammatory cytokines and whether, independent of their social anxiety, patients with psoriasis show a higher release of neuroinflammatory parameters than skin-healthy controls.

Own preparatory work

In a recent study of the European Society for Dermatoloy and Psychiatry (ESDAP), in which Dr. Schut was involved together with researchers from 13 different European countries and whose study center is located in Giessen (Head: PD Dr. Jörg Kupfer, cooperation partner in this project), it was shown that itching is a very common symptom in patients with psoriasis

(Schut et al., 2019). Further data from this project illustrate that the presence of itching is significantly related to psychological distress in skin patients (Dalgard et al., 2019).

In our own experimental work, also together with American scientists, we were also able to identify different psychological variables as predictors of itch/scratch behavior in patients with chronic itch (Schut et al., 2014a, 2015a, 2016, 2018). In psoriasis patients specifically, a positive association was found between public self-attention, the tendency to pay attention to what others think of one, and induced itch (Schut et al., 2015a). Furthermore, we found that psoriasis patients exhibit more skin-related disgust and shame than skin-healthy controls (Lahousen et al., 2016). The results of the latter two studies are of great importance for the planned research project, as they show that psoriasis patients have particularly intense feelings of shame due to their skin and that their itch sensation is partly determined by what others think of them. From these results it can be deduced that patients with great social anxiety, possibly caused by feelings of shame and stigmatization, could also experience more intense itching.

The next step is to investigate whether this relationship is mediated by the release of neuroinflammatory cytokines. The main applicant was able to gain experience in the analysis of neuroinflammatory cytokines in the context of her doctoral thesis (Schut, 2013). The cooperation with the Psychoneuroimmunology Laboratory of the Department of Psychosomatics and Psychotherapy (Head: Prof. Eva Peters), which has expertise in the analysis of neuroinflammatory parameters and the application of the suction blister method, enables the valid investigation of neuroinflammatory parameters. The planned statistical methods follow already proven analyses (Schut et al., 2014b; 2015b).

Questions and hypotheses

This study will answer the following questions and test hypotheses:

Research Question 1: Does social anxiety represent a predictor of experienced itch intensity in psoriasis patients?

The first hypothesis (H1) is: Social anxiety represents a significant predictor of experienced itch intensity in psoriasis patients.

Research Question 2: Do neuroinflammatory parameters (IL-6, IL-17, substance P) represent mediators of the association between social anxiety and itch intensity in psoriasis patients?

The second hypothesis (H2) is that the neuroinflammatory parameters IL-6, IL-17, and substance P mediate the association between social anxiety and itch intensity in psoriasis patients.

Secondary Question 1: Do high- and low-anxious skin-healthy subjects differ in the release of the neuroinflammatory parameters IL-6, IL-17, and substance P?

Secondary hypothesis 1 (NH1): High- and low-anxious skin-healthy subjects differ in the release of the neuroinflammatory parameters IL-6, IL-17, and substance P.
Secondary question 2: Do psoriasis patients and skin-healthy subjects differ in the release of the neuroinflammatory parameters IL-6, IL-17 and substance P?
Secondary hypothesis (NH2): Skin-healthy subjects differ from psoriasis patients in the release of the neuroninflammatory parameters IL-6, IL-17 and substance P.
Secondary question 3: Do psoriasis patients and skin-healthy subjects differ in terms of social anxiety?
Secondary hypothesis (NH3): Skin-healthy subjects differ from psoriasis patients with respect to social anxiety.

Definition of the collective and characteristics of the patients/subjects to be included in the study.

Male and female subjects aged 18-65 years who have a sufficient knowledge of the German language to be able to answer the questionnaires without difficulty will be included. Half of the subjects are skin-healthy subjects and the other half are patients with clinically diagnosed psoriasis, in whom the skin disease has been present for at least six months. Subjects with another (skin) disease associated with itching are not allowed to participate in the research project. Similarly, subjects will be excluded from the study if a body fluid transmissible disease is present. The presence of transmissible diseases is inquired about and recorded in great detail. Subjects who are treated with biologics, i.e. drugs that are produced from cell cultures and interfere with the patient's own immune system, are also excluded from participation in Study 2. In addition, to participate in Study 2, it is necessary to have been classified by the SIAS as either high or low anxiety.

Procedure for the recruitment of patients and study procedure

The research project can be divided into two studies:
Study 1: 250 patients with clinically diagnosed psoriasis will be made aware of the study in dermatological specialist practices in the vicinity of Giessen and through notices. Furthermore, 250 skin-healthy test persons will be included in the study parallel to the psoriasis patients. They will be recruited via notices (e.g. in supermarkets, by addressing them on the campus of the JLU, etc.). All subjects will be recruited by a wiss. All subjects will be informed about the study by a research assistant and/or a student assistant and will receive the consent form. After consent, subjects will complete the questionnaire set to determine the expression of social anxiety and itch intensity and to record the control variables (see below). Subjects will receive €10 for participating in Study 1 and will be asked to leave their contact information to

be contacted if they are interested in Study 2 and are eligible. The data will be stored pseudonymously (see below). Study 2: Patients and skin-healthy subjects will be selected from the pool of participants in Study 1 according to their level of social anxiety (see inclusion criteria). They will be informed about Study 2 and the suction blister method in a telephone conversation. In addition, the inclusion and exclusion criteria (see below) will be clarified by telephone. For inclusion in Study 2, 64 skin-healthy subjects and 64 psoriasis patients will come to the laboratory rooms of the Institute of Medical Psychology on two dates. They will receive an expense allowance of 100 € for participation in study 2. On the first examination date the test persons are accustomed to the method of the suction blister by being informed in detail about the procedure of the method, by having a look at the suction pump and by putting on the plexiglass suction chamber for 15 minutes and applying negative pressure. This appointment is only for habituation to this method and not for recording the neuroinflammatory parameters. This takes place on a second examination appointment and lasts a maximum of 3.5 hours. At the end of the first examination appointment, the subjects sign the consent form for study 2. At the beginning of the second examination appointment, they first complete questionnaires (recording of control variables, recording of social anxiety and itch; see below). Subsequently, the subjects will be subjected to the suction blister method and thus tissue fluid will be obtained in order to determine the neuroinflammatory parameters therein (see also below).

<u>Which characteristics, measurands (variables) are observed or determined?</u>

1) Social anxiety

Social anxiety is assessed in both Study 1 and Study 2 using the same questionnaires. Two social anxiety disorder scales (SOZAS; von Consbruch et al., 2016) are used. They are the Social Interaction Anxiety Scale (SIAS) and the Social Phobia Scale (SPS). These two measurement instruments, validated in the German language, each include 20 questions answered on a 5-point scale. While the SIAS measures interaction anxiety, the SPS measures anxiety in evaluative situations. The items in both questionnaires do not refer to a specific time period. In this study, subjects are defined as 26highly socially anxious (HSA) according to the cut-off value determined by Stangier et al, (1999) with SIAS scores ≥ 26. Subjects with a percentile rank ≤ 25 in Study 1 are considered low socially anxious (LSA).

2) Itching intensity

In study 1, after recording control variables and social anxiety, both current itch intensity and itch intensity within the last 24 hours before the examination appointment are recorded by visual analog scale (VAS 0-10) with the two poles "no itch" (0) and "worst imaginable itch" (10). In addition, PS patients are asked about the presence of chronic itch and the current itch intensity is surveyed in relation to the worst itch intensity experienced due to psoriasis. For this

purpose, patients are asked to relate their current itch to the maximum itch they experienced due to psoriasis. They are asked to indicate whether the current itch is as severe, minimally better, somewhat better, significantly better, or very significantly better. In Study 2, at the beginning of the second examination appointment, the current itch is determined as a baseline value, also via the use of the VAS described above. During the course of application of the suction blister method, itch intensity is also recorded at 30-minute intervals by VAS (0-10) at a maximum of six measurement time points until the formation of the suction blisters and subsequent fluid removal.

3) Neuroinflammatory parameters

The concentration of the neuroinflammatory parameters IL-6, IL-17, and substance-P will be determined in tissue fluid obtained by the suction blister method (see below) using ELISA assays (IBL International, Hamburg, Germany) according to modified manufacturer's recommendations. A comprehensive panel of mediators of innate and learned immunity could be determined in future, further investigations by cytometric bead array analysis (Bender MedSystems, eBioscience, Frankfurt, Germany).

The suction blister method used in Study 2 was developed more than 50 years ago (Kiistala, 1968) and allows painless ablation of the upper layers of the epidermis from the dermis, forming a blister above the basement membrane and causing no injury to the underlying structures. Thus, there is no pain, bleeding or visible subsequent healing (e.g. scarring). The suction blisters are generated by a constant negative pressure of 200-300 mmHG, by means of a finely adjustable suction pump and a plexiglass suction chamber specially made for this purpose from a workshop of the Charité with 3 circular, 8 mm diameter openings as shown in Figure 2. Negative pressure is applied for 1-3 hours until the blister develops. This process results in the transfer of interstitial fluid from the epidermis and dermis into the blister cavity, including intercellularly contained proteins and other messengers such as neurotransmitters, neuropeptides, as well as cytokines (Niedzwiecki et al., 2018; Mias et al., 2018; Myles et al., 2018; Tobin et al., 2000). Thus, a variety of neuroinflammatory parameters are measurable in the interstitial fluid. The fluid is aspirated sterilely from the blister using an insulin syringe, transferred to an Eppendorf tube, centrifuged, and the supernatant stored at -80°C until analysis of the neurotransmitters it contains. For possible further molecular biological analyses of mRNA expression or epigenetic methylation patterns, the centrifuged cells of the immune system as well as the blister roof will also be stored at -80°C. The supernatant will be transferred into an Eppendor tube. Here, we refrain from explicitly provoking an immune response in advance as in other studies (Holm et al., 2018), but instead examine the natively present cells under disease and different stress conditions (high vs. low social anxiety). The suction blister is generated on non-lesional skin of the patient's forearm. The blister is induced to be at least 3 cm away from the nearest lesional skin site.

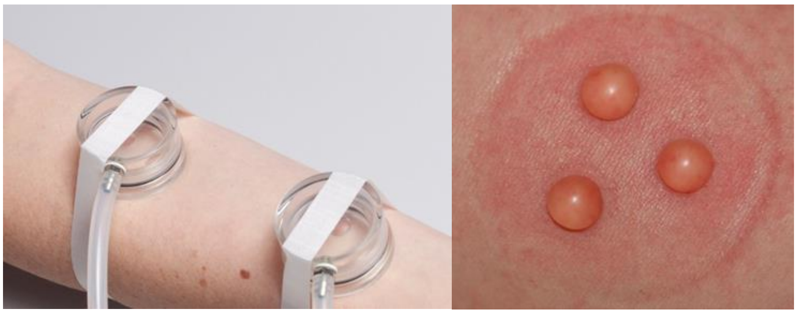

Figure 2. Application of the minimally invasive suction blister method, in which tissue fluid is suctioned into blisters by negative pressure and can be removed painlessly from there (image source: http://www.sit-skin.de/services/suction-blister-method).

What are the variables essential to answer the question?

- Social anxiety (studies 1 and 2)
- Itch intensity (studies 1 and 2)
- Neuroinflammatory parameters IL-6, IL-17 and substance P (study 2).

Which variables should be used to obtain additional information

As control variables, personal variables (age, gender, BMI, smoking, use of oral contraceptives, and social status operationalized via the highest school degree attained) and the self-assessed severity of psoriasis during the last 24 hours before the start of the study via the SA-PASI (Feldman et al., 1996) will be collected in study 1 and 2. In addition, in study 2, current mental health status is assessed using the short form of the German Health Questionnaire for Patients (PHQ-D; Gräfe et al., 2004) and general and momentary anxiety experience is assessed using the State-Trait Anxiety Inventory (STAI; Laux et al., 1981). Also, patients in whom the release of neuroinflammatory cytokines can be assumed to be altered by the use of biologics will be excluded from participation in Study 2 (see below). Gender will be stratified as much as possible within the "HSA" and "LSA" subgroups. If it is the case that the sex ratio in the HSA and/or LSA group is not balanced, sex will enter the analyses as a covariate. In addition, the time it takes for the suction blister to form on the skin is collected as a control variable.

Which disturbance variables can influence the result

There are no known disturbance variables that can influence the result that are not recorded.

Biometric evaluation planning and sample size considerations.

To answer research question 1, a linear hierarchical regression is performed, with control variables in the first step and social anxiety (measured via the SIAS and SPS) as potential predictor variables in the second step. Criterion variable represents itch intensity.

To answer research question 2, a mediation analysis (Baron & Kenny, 1986; Preacher & Hayes, 2008) will be conducted using the SPSS macro "Process". Included in the analysis will be control variables and social anxiety as predictor variables, neuroinfammatory parameters as mediators, and itch intensity as a criterion variable. A calculation of the required sample size using G-Power (Faul et al., 2009) to answer the central research question 2 revealed that to perform the mediation analysis, 64 patients with psoriasis (n=32 with low and n=32 with high social anxiety (SIAS score ≥ 26) must be studied to detect small to medium effects (f^2=0.10). Small to moderate effects can be assumed because, on the one hand, only extreme groups are examined and, on the other hand, small to moderate correlations between anxiety and itch intensity in psoriasis patients (e.g., Mrowietz et al., 2015) and between neuroinflammatory parameters and itch intensity have been found in previous studies (Konda et al., 2015). In order to include n=64 patients in Study 2, 250 psoriasis patients will be screened in Study 1. From this pool, all patients who have high social anxiety scores (value in SIAS ≥ 26 (cut-off value)) and those who have low social anxiety scores (PR ≤ 25) will be approached and motivated to participate in Study 2. The targeting of the large number of subjects is necessary to include n=64 subjects in Study 2. Single-factor analyses of covariance are calculated to answer the subsidiary questions.

If interim evaluation is provided

Interim evaluations are not provided.

If design changes should/could be made

Design changes are not foreseen.

Ethics

Subjects participate in the planned project on a voluntary basis. They will receive 10 € volunteer fee for participation in study 1. Their motivation is also to contribute to the improvement of knowledge about factors that contribute to itch intensity in psoriasis patients. By completing the questionnaires, they are not exposed to any risks. There are also no risks associated with the use of the suction blister method. As described above, it is a non-pain inducing method that does not cause scarring. It is only possible that a slight, transient itching is produced by the method, which is also surveyed.

Privacy

The provisions of the data protection act are strictly followed. The questionnaires are entered in pseudonymized form. The questionnaires are therefore only provided with an individual pseudonymized code and are kept separately from the consent forms There will, however, be

assignment lists from which it is possible to draw conclusions from the collected data to the test person. This is necessary in that if high or low social anxiety is identified in the questionnaire study (Study 1), subjects will be contacted again to motivate them to participate in Study 2. After completion of the questionnaires, they will be stored separately from the consent form in locked cabinets at the Institute of Medical Psychology, Justus Liebig University Giessen, for a period of 10 years, as they are primary scientific data. For statistical analysis, the data are entered into SPSS 26 (IBM Corp, 2016). Afterwards, they will be destroyed. The consent forms will also be destroyed after the ten-year retention period has been met. The subjects can revoke their consent to participate in the study until the questionnaires are handed in, without giving reasons. This will not result in any disadvantages for them.

Measures to ensure patient safety

Participation in the study does not pose any psychological or physical risks to the subjects.

Quality management

The questionnaires are evaluated according to the guidelines in the test manuals.

Publication

The publication of the results is planned in the form of a dissertation. In addition, the results will be published in international journals in the field of psychosomatics and dermatology.

If the study protocol is to be published or the study is to be registered

It is planned to register this study with the German Register of Clinical Trials (DRKS), as many journals require a Clinical Trial Number for publication.

Literature

1. Alpsoy E, Polat M, FettahlıoGlu-Karaman B, Karadag AS, Kartal-Durmazlar P, YalCın B, Emre S, Didar-Balcı D, Bilgic-Temel A, Arca E, Koca R, Gunduz K, Borlu M, Ergun T, Dogruk-Kacar S, Cordan-Yazici A, Dursun P, BilgiC O, Gunes-Bilgili S, Sendur N, Baysal O, Halil-Yavuz I, Yagcioglu G, Yilmaz E, Kavuzlu U, Senol Y. Internalized stigma in psoriasis: A multicenter study. J Dermatol. 2017;44:885-891.
2. Augustin M, Reich K, Glaeske G, Schaefer I, Radtke M. Co-morbidity and age-related prevalence of psoriasis: analysis of health insurance data in Germany. Acta Derm Venereol. 2010;90:147-151.
3. Baron RM, Kenny DA. The moderator-mediator variable distinction in social psychological research: conceptual, strategic, and statistical considerations. J Pers Soc Psychol. 1986;51:1173-82.
4. Connor CJ, Liu V, Fiedorowicz JG. Exploring the physiological link between psoriasis and mood disorders. Dermatol Res Pract. 2015:409637. doi: 10.1155/2015/409637
5. Dalgard FJ, Svensson Å, Halvorsen JA, Gieler U, Schut C, Tomas-Aragones L, Lien L, Poot F, Jemec GBE, Misery L, Szabo C, Linder D, Sampogna F, Spillekom-van Koulil S, Balieva F, Szepietowski JC, Lvov A, Marron SE, Altunay IK, Finlay AY, Salek S, Kupfer J. Itch and mental health in dermatological patients across Europe: a cross sectional study in 13 countries. J Invest Dermatol. 2019; doi: 10.1016/j.jid.2019.05.034
6. Faul F, Erdfelder E, Buchner A, Lang AG. Statistical power analyses using G*Power 3.1: Tests for correlation and regression analyses. Behav Res Methods. 2009; 41:1149-1160.
7. Feldman SR, Fleischer AB, Reboussin DM, Rapp SR, Exum ML, Clark AR, Nurre L. The self-administered psoriasis area and severity index is valid and reliable. J Invest Dermatol. 1996;106:183-186.
8. Globe D, Bayliss MS, Harrison DJ. The impact of itch symptoms in psoriasis: results from physician interviews and patient focus groups. Health Qual Life Outcomes. 2009 Jul 6;7:62. doi: 10.1186/1477-7525-7-62
9. Gräfe K, Zipfel S, Herzog W, Löwe B. Screening psychischer Störungen mit dem "Gesundheitsfragebogen für Patienten (PHQ-D)" Diagnostica 2004; 50:171-181.
10. Holm LL, Vukmanovic-Stejic M, Blauenfeldt T, Benfield T, Andersen P, Akbar AN, Ruhwald M. A Suction Blister Protocol to Study Human T-cell Recall Responses In Vivo. J Vis Exp. 2018;138.e57554, doi:10.3791/57554.
11. Kiistala U. Suction blister device for separation of viable epidermis from dermis. J Invest Dermatol. 1968; 50:129-137.
12. Konda D, Chandrashekar L, Rajappa M, Kattimani S, Thappa DM, Ananthanarayanan PH. Serotonin and interleukin-6: Association with pruritus severity, sleep quality and depression severity in prurigo nodularis. Asian J Psychiatr. 2015;17:24-28.
13. Lahousen T, Kupfer J, Gieler U, Hofer A, Linder MD, Schut C. Differences Between Psoriasis Patients and Skin-healthy Controls Concerning Appraisal of Touching, Shame and Disgust. Acta Derm Venereol. 2016;96:78-82.
14. Laux L, Glanzmann P, Schaffner P, Spielberger CD. STAI – Das State-Trait Angstinventar. 1981; Hogrefe: Göttingen.
15. Leff Gelman P, Mancilla-Herrera I, Flores-Ramos M, Saravia Takashima MF, Cruz Coronel FM, Cruz Fuentes C, Pérez Molina A, Hernández-Ruiz J, Silva-Aguilera FS, Farfan-Labonne B, Chinchilla-Ochoa D, Garza Morales S, Camacho-Arroyo I. The cytokine profile of women with severe anxiety and depression during pregnancy. BMC Psychiatry. 2019;19:104. doi: 10.1186/s12888-019-2087-6
16. Liang SE, Cohen JM, Ho RS. Psoriasis and suicidality: a review of the literature. Dermatol Ther. 2019;32:e12771. doi: 10.1111/dth.12771
17. Liu Y, Ho RC, Mak A. The role of interleukin (IL)-17 in anxiety and depression of patients with rheumatoid arthritis. Int J Rheum Dis. 2012;15:183-187.
18. Mias C, Le Digabel J, Filiol J, Gontier E, Gravier E, Villaret A, Nocera T, Questel E, Rossi AB, Redoulès D, Josse G. Visualization of dendritic cells' responses in atopic dermatitis: preventive effect of emollient. Exp Dermatol. 2018;27:374-377.

19. Mrowietz U, Chouela EN, Mallbris L, Stefanidis D, Marino V, Pedersen R, Boggs RL. Pruritus and quality of life in moderate-to-severe plaque psoriasis: post hoc exploratory analysis from the PRISTINE study. J Eur Acad Dermatol Venereol. 2015;29:1114-1120.
20. Muñoz M, Coveñas R. Involvement of substance P and the NK-1 receptor in human pathology. Amino Acids. 2014;46:1727-1250.
21. Myles IA, Anderson ED, Earland NJ, Zarember KA, Sastalla I, Williams KW, Gough P, Moore IN, Ganesan S, Fowler CJ, Laurence A, Garofalo M, Kuhns DB, Kieh MD, Saleem A, Welch PA, Darnell DA, Gallin JI, Freeman AF, Holland SM, Datta SK. TNF overproduction impairs epithelial staphylococcal response in hyper IgE syndrome. J Clin Invest. 2018;128:3595-3604.
22. Nattkemper LA, Tey HL, Valdes-Rodriguez R, Lee H, Mollanazar NK, Albornoz C, Sanders KM, Yosipovitch G. The Genetics of Chronic Itch: Gene Expression in the Skin of Patients with Atopic Dermatitis and Psoriasis with Severe Itch. J Invest Dermatol. 2018;138:1311-1317.
23. Niedzwiecki MM, Samant P, Walker DI, Tran V, Jones DP, Prausnitz MR, Miller GW. Human Suction Blister Fluid Composition Determined Using High-Resolution Metabolomics. Anal Chem. 2018;90:3786-3792.
24. Preacher KJ, Hayes AF. Asymptotic and resampling strategies for assessing and comparing indirect effects in multiple mediator models. Behav Res Methods. 2008;40:879-891.
25. Sanders KM, Akiyama T. The vicious cycle of itch and anxiety. Neurosci Biobehav Rev, 2018; 87:17-26.
26. Sanders KM, Sakai K, Henry TD, Hashimoto T, Akiyama, T. A subpopulation of amygdala neurons mediates the affective component of Itch. J Neurosci. 2019;39:3345-3356.
27. Schut C. Stress management in atopic dermatitis: psychophysiological effects. 2013. dissertation. http://geb.uni-giessen.de/geb/volltexte/2013/9914/
28. Schut C, Bosbach S, Gieler U, Kupfer, J. Personality traits, depression and itch in patients with atopic dermatitis in an experimental setting: a regression analysis. Acta Derm Venereol. 2014a; 94: 20-25.
29. Schut C, Felsch A, Zick C, Hinsch KD, Gieler U, Kupfer J. Role of illness representations and coping in patients with atopic dermatitis: a cross-sectional study. J Eur Acad Dermatol Venereol. 2014b;28:1566-1571.
30. Schut C, Muhl S, Reinisch K, Claßen A, Jäger R, Gieler U, Kupfer J. Agreeableness and self-consciousness as predictors of induced scratching and itch in patients with psoriasis. Int J Behav Med. 2015a;22:726-734.
31. Schut C, Weik U, Tews N, Gieler U, Deinzer R, Kupfer J. Coping as mediator of the relationship between stress and itch in patients with atopic dermatitis: a regression and mediation analysis. Exp Dermatol. 2015b;24:148-150.
32. Schut C, Rädel A, Frey L, Gieler U, Kupfer, J. Role of personality and expectations for itch and scratching induced by audiovisual itch stimuli. Eur J Pain. 2016;20:14-18.
33. Schut C, Reinisch K, Classen A, Andres S, Gieler U, Kupfer, J. Agreeableness as Predictor of Induced Scratching in Patients with Atopic Dermatitis: A Replication Study. Acta Derm Venereol. 2018; 98:32-37.
34. Schut C, Dalgard FJ, Halvorsen JA, Gieler U, Lien L, Aragones LT, Poot F, Jemec GBE, Misery L, Kemény L, Sampogna F, van Middendorp H, Balieva F, Linder D, Szepietowski JC, Lvov A, Marron SE, Altunay IK, Finlay AY, Salek S, Kupfer J. Occurrence, Chronicity and Intensity of Itch in a Clinical Consecutive Sample of Patients with Skin Diseases: A Multi-centre Study in 13 European Countries. Acta Derm Venereol. 2019; 99:146-151.
35. Sommer R, Augustin M, Mrowietz U, Topp J, Schäfer I, von Spreckelsen R. Perception of stigmatization in people with psoriasis-qualitative analysis from the perspective of patients, relatives and healthcare professionals. Dermatologist. 2019;70:520-526.
36. Stangier U, Heidenreich T, Berardi A, Golbs U, Hoyer J. Die Erfassung sozialer Phobie durch die Social Interaction Anxiety Scale (SIAS) und die Social Phobia Scale (SPS). Zeitschrift für Klinische Psychologie 1999;28:28-36.
37. Szepietowski JC, Reich A. Pruritus in psoriasis: An update. Eur J Pain. 2016;20:41-6.

38. Théréné C, Brenaut E, Barnetche T, Misery L. Efficacy of Systemic Treatments of Psoriasis on Pruritus: A Systemic Literature Review and Meta-Analysis. J Invest Dermatol. 2018;138:38-45.
39. Tobin DJ, Swanson NN, Pittelkow MR, Peters EM, Schallreuter KU. Melanocytes are not absent in lesional skin of long duration vitiligo. J Pathol. 2000;191:407-416.
40. von Consbruch K, Stangier U, Heidenreich T. SOZAS - Social anxiety disorder scales. 2016; Hogrefe: Göttingen.
41. Wang X-D, Yang G, Bai Y, Feng Y-P, Li H. The behavioral study on the interactive aggravation between pruritus and depression. Brain Behav. 2018;8:e00964. doi: 10.1002/brb3.964
42. Zachariae R, Lei U, Haedersdal M, Zachariae C. Itch severity and quality of life in patients with pruritus: preliminary validity of a Danish adaptation of the itch severity scale. Acta Derm Venereol. 2012;92:508-14.
43. Zeidler C, Pereira MP, Huet F, Misery L, Steinbrink K, Ständer S. Pruritus in autoimmune and inflammatory dermatoses. Front Immunol. 2019;10:1303. doi: 10.3389/fimmu.2019.01303
44. Zhao X, Yu C, Ye F, Wang YG, Mei QY, Ma Q, Cui WG, Zhou WH. Chronic itch impairs mood and HPA axis function in mice: modulation by CRFR1 antagonist. Pain 2018;159: 2201-2213
