# Supplementary material for: Study protocol: Neuro-inflammatory parameters as mediators of the relationship between social anxiety and itch intensity: A cross-sectional, controlled laboratory study in patients with psoriasis and healthy skin controls
Source: PLoS One. 2023 Mar 16;18(3):e0281989. doi: 10.1371/journal.pone.0281989 (PMC10019658; doi:10.1371/journal.pone.0281989)
Supplement: S2 File — (DOCX) [file pone.0281989.s003.docx]

Ethikantrag Anlage D:

Genaues klinisches Studienprotokoll mit detaillierter Biometrie

**Titel: Neuro-Inflammatorische Parameter als Mediatoren des Zusammenhangs zwischen sozialer Angst und Juckreizintensität bei Psoriasis-Patienten**

**Ziel und Motivation der Studie**

Psoriasis ist eine chronisch-entzündliche Hauterkrankung, die bei der Mehrheit der Patienten mit Juckreiz einerseits und Stigmatisierungsgefühlen andererseits einhergeht. Stigmatisierung führt zu sozialer Angst mit Vermeidungsverhalten und sozialem Rückzug. Ein möglicher pathogenetischer Zusammenhang zwischen sozialer Angst und Juckreiz ist herleitbar, da Psoriasis-Patienten über erhöhte Angstwerte berichten und Angst bei Psoriasis-Patienten signifikant positiv mit Juckreiz-Intensität korreliert. Dieser Zusammenhang zwischen sozialer Angst und Juckreiz wurde bislang aber nicht untersucht. Gerade die soziale Angst kann dazu führen, dass eine physiologische Reaktion auf soziale Belastungssituationen wie die Ausschüttung neuroinflammatorischer Parameter im Übermaß in Gang gesetzt wird und Juckreiz verstärkt. IL-6, IL-17 und Substanz-P sind Parameter, die bei erhöhtem Angsterleben und Juckreiz eine Rolle spielen und sollen daher als Mediatoren des Zusammenhangs zwischen sozialer Angst und Juckreiz in diesem Forschungsprojekt untersucht werden (Abb. 1). Ziel der ersten Studie ist es, bei Psoriasis-Patienten den direkten Zusammenhang zwischen sozialer Angst und Juckreizintensität zu bestimmen. In einer zweiten Studie werden bei ausgewählten Patienten aus Studie 1 neuroinflammatorische Parameter mittels der minimal-invasiven Suction-Blister-Technik bestimmt, um zu untersuchen, ob diese signifikante Mediatoren im Zusammenhang zwischen sozialer Angst und Juckreiz darstellen.

**Neuro-**

**inflammatorische Parameter**

***Psoriasis***

**Juckreiz**

**Soziale Angst**

Abbildung 1. Ziel der Studie ist es, den mediierenden Effekt von neuroinflammatorischen Parametern im Zusammenhang zwischen sozialer Angst und Juckreiz bei Psoriasis zu untersuchen.

**Stand der Forschung**

Bei der chronisch-entzündlichen Hauterkrankung Psoriasis, die rund 2.5% der deutschen Bevölkerung betrifft, stellt Juckreiz mit einer Prävalenz von 60-90% ein häufiges Symptom dar, das als äußerst unangenehm und belastend empfunden wird (Globe et al., 2009; Augustin et al., 2010; Szepietowksi & Reich, 2016; Schut et al., 2019). Psoriasis-Patienten berichten von Stigmatisierung (Alpsoy et al., 2017; Sommer et al., 2019), welche durch das veränderte Erscheinungsbild der Haut und Kratzläsionen erklärbar ist. In mehreren Studien zeigt sich ein positiver Zusammenhang zwischen der Juckreizintensität und Angst in dieser Patientengruppe (z.B. Mrowietz et al. 2015; Zachariae et al., 2012). Auch im Tiermodell zeigt sich, dass bei Mäusen, bei denen unvorhersehbarer Stress über 28 Tage appliziert wurde, mehr spontanes Kratzverhalten auftrat als in der Kontrollgruppe (Wang et al., 2018). Darüber hinaus wurde beobachtet, dass akuter (Sanders et al., 2019) und chronischer Juckreiz (Zhao et al., 2018; Wang et al., 2019) bei Mäusen zu Verhaltensweisen führt, die auf eine ängstliche Symptomatik hinweisen.

Man geht von einem Juckreiz-Angst-Zirkel aus (Sanders & Akiyama, 2018), welchen es bei Patienten mit chronischem Juckreiz zu durchbrechen gilt. Besondere Aufmerksamkeit muss in diesem Zirkel der sozialen Angst als einer Facette der Angst gebühren, welche zu Vermeidungsverhalten und sozialem Rückzug führt. Diese können so weitere typische psychische Probleme wie Depressionen und Suizidalität nach sich ziehen, die gehäuft bei Psoriasis-Patienten beobachtet werden (Liang et al., 2019). Der sehr wichtige Zusammenhang von sozialer Angst und Juckreiz bei Psoriasis-Patienten wurde bislang nicht erfasst und wird deswegen Bestandteil des Forschungsprojektes sein (Fragestellung 1).

Aktuelle Forschung legt nahe, dass IL-6, IL-17 und Substanz P bei der Juckreizentstehung generell und die letztgenannten besonders bei der Psoriasis eine Rolle spielen (z.B. Konda et al., 2015; Théréné et al., 2018; Zeidler et al., 2019; Nattkemper et al., 2018). Diese neuroinflammatorischen Parameter werden andererseits auch mit Angst in Zusammenhang gebracht (z.B. Muñoz & Coveñas, 2014; Liu et al., 2012; Leff Gelman et al., 2019). Der Link zwischen Angst und Psoriasis-Symptomen könnte demnach über die Ausschüttung neuroinflammatorischer Zytokine vermittelt werden (Connor et al., 2015). Jedoch wurde dies bislang noch nicht untersucht und steht deswegen im Fokus des geplanten Projekts (Fragestellung 2).

Als Nebenfragestellung wird untersucht, ob sich sozial niedrig und hoch ängstliche hautgesunde Kontrollen in ihrer lokalen Ausschüttung neuroinflammatorischer Zytokine unterscheiden und ob unabhängig von ihrer sozialen Ängstlichkeit Patienten mit Psoriasis eine höhere Ausschüttung neuroinflammatorischer Parameter aufweisen als hautgesunde Kontrollen.

**Eigene Vorarbeiten**

In einer aktuellen Studie der European Society for Dermatoloy and Psychiatry (ESDAP), an der Dr. Schut gemeinsam mit Forschern 13 unterschiedlicher europäischer Länder beteiligt war und deren Studienzentrum in Gießen liegt (Leitung: PD Dr. Jörg Kupfer, Kooperationspartner in diesem Projekt) konnte gezeigt werden, dass Juckreiz ein sehr häufiges Symptom bei Patienten mit Psoriasis darstellt (Schut et al., 2019). Weitere Daten aus diesem Projekt verdeutlichen, dass das Vorhandensein von Juckreiz signifikant mit der psychischen Belastung bei Hautpatienten zusammenhängt (Dalgard et al., 2019).

In eigenen experimentellen Arbeiten konnten wir, auch gemeinsam mit amerikanischen Wissenschaftlern, bei Patienten mit chronischem Juckreiz zudem unterschiedliche psychologische Variablen als Prädiktoren für Juckreiz/ Kratzverhalten identifizieren (Schut et al., 2014a, 2015a, 2016, 2018). Bei Psoriasis-Patienten im Speziellen ergab sich ein positiver Zusammenhang zwischen öffentlicher Selbstaufmerksamkeit, der Neigung darauf zu achten, was andere von einem denken, und induziertem Juckreiz (Schut et al., 2015a). Weiterhin fanden wir, dass Psoriasis-Patienten mehr hautbezogenen Ekel und Scham aufweisen als hautgesunde Kontrollen (Lahousen et al., 2016). Die Ergebnisse der beiden zuletzt genannten Studien sind für das geplante Forschungsprojekt von großer Wichtigkeit, da sie zeigen, dass Psoriasis-Patienten aufgrund ihrer Haut besonders intensive Schamgefühle haben und ihr Juckreizempfinden davon mitbestimmt wird, was andere von ihnen denken. Aus diesen Ergebnissen lässt sich ableiten, dass Patienten mit großer sozialer Angst, evtl. durch Scham und Stigmatisierungsgefühle hervorgerufen, auch intensiveren Juckreiz empfinden könnten.

Im nächsten Schritt gilt es zu untersuchen, ob dieser Zusammenhang über die Ausschüttung neuroinflammatorischer Zytokine vermittelt wird. Erfahrungen in der Analyse neuroinflammatorischer Zytokine konnte die Hauptantragstellerin im Rahmen ihrer Promotionsarbeit sammeln (Schut, 2013). Die Kooperation mit dem Psychoneuroimmunologie-Labor der Klinik für Psychosomatik und Psychotherapie (Leitung: Prof. Eva Peters), das über Expertise in der Analyse neuroinflammatorischer Parameter und die Anwendung der Suction Blister Methode verfügt, ermöglicht die valide Untersuchung der neuroinflammatorischen Parameter. Die geplanten statistischen Methoden folgen bereits erprobten Analysen (Schut et al., 2014b; 2015b).

**Fragestellungen und Hypothesen**

Mit dieser Studie werden folgende Fragestellungen beantwortet und Hypothesen geprüft:

Fragestellung 1: Stellt soziale Angst einen Prädiktor für erlebte Juckreizintensität bei Psoriasis-Patienten dar?

Die erste Hypothese (H1) lautet: Soziale Angst stellt einen signifikanten Prädiktor erlebter Juckreizintensität bei Psoriasis-Patienten dar.

Fragestellung 2: Stellen neuroinflammatorische Parameter (IL-6, IL-17, Substanz P) Mediatoren des Zusammenhangs von sozialer Angst und Juckreizintensität bei Psoriasis-Patienten dar?

Die zweite Hypothese (H2) lautet: Die neuroinflammatorischen Parameter IL-6, IL-17 und Substanz P mediieren den Zusammenhang zwischen sozialer Angst und Juckreizintensität bei Psoriasis-Patienten.

Nebenfragestellung 1: Unterscheiden sich hoch und niedrig ängstliche hautgesunde Probanden in der Ausschüttung der neuroinflammatorischen Parameter IL-6, IL-17 und Substanz P?

Nebenhypothese 1 (NH1): Hoch und niedrig ängstliche hautgesunde Probanden unterscheiden sich in der Ausschüttung der neuroinflammatorischen Parameter IL-6, IL-17 und Substanz P.

Nebenfragestellung 2: Unterscheiden sich Psoriasis-Patienten und hautgesunde Probanden in der Ausschüttung der neuroinflammatorischen Parameter IL-6, IL-17 und Substanz P?

Nebenhypothese (NH2): Hautgesunde Probanden unterscheiden sich von Psoriasis-Patienten in der Ausschüttung der neuroninflammatorischen Parameter IL-6, IL-17 und Substanz P.

Nebenfragestellung 3: Unterscheiden sich Psoriasis-Patienten und hautgesunde Probanden hinsichtlich sozialer Angst?

Nebenhypothese (NH3): Hautgesunde Probanden unterscheiden sich von Psoriasis-Patienten hinsichtlich sozialer Angst.

**Definition des Kollektivs und der Eigenschaften der Patienten/ Probanden, die in die Studie aufgenommen werden sollen**

Es werden männliche und weibliche Probanden im Alter von 18-65 Jahren eingeschlossen, die die deutsche Sprache ausreichend gut beherrschen, um die Fragebögen problemlos beantworten zu können. Bei den Probanden handelt es sich zur Hälfte um hautgesunde Probanden und zur anderen Hälfte um Patienten mit klinisch diagnostizierter Psoriasis, bei denen die Hauterkrankung seit mindestens sechs Monaten vorliegt. Probanden, bei denen eine andere mit Juckreiz assoziierte (Haut-)erkrankung im Fokus steht, dürfen nicht an dem Forschungsprojekt teilnehmen. Ebenso werden Probanden von der Studie ausgeschlossen, sofern eine durch Körperflüssigkeiten übertragbare Erkrankung vorliegt. Das Vorhandensein übertragbarer Erkrankungen wird sehr genau erfragt und protokolliert. Auch sind Probanden, die mit Biologika, also Medikamenten, die aus Zellkulturen hergestellt werden und in das eigene Immunsystem eingreifen, behandelt werden, von der Teilnahme an Studie 2 ausgeschlossen. Zudem ist es für die Teilnahme an Studie 2 notwendig, durch den SIAS als entweder hoch- oder niedrig ängstlich klassifiziert worden zu sein.

**Verfahren zur Rekrutierung der Patienten und Studienablauf**

Das Forschungsprojekt lässt sich in zwei Studien unterteilen:

Studie 1: 250 Patienten mit klinisch diagnostizierter Psoriasis werden in dermatologischen Facharztpraxen im Umkreis von Gießen und durch Aushänge auf die Studie aufmerksam gemacht. Weiterhin werden 250 hautgesunde Probanden parallel zu den Psoriasis-Patienten in die Studie eingeschlossen. Sie werden über Aushänge (z.B. in Supermärkten, durch Ansprache auf dem Campus der JLU etc. rekrutiert). Alle Probanden werden von einem wiss. Mitarbeiter und/oder einer studentischen Hilfskraft über die Studie aufgeklärt und erhalten anschließend die Einwilligungserklärung. Nach Einwilligung füllen die Probanden das Fragebogenset zur Bestimmung der Ausprägung sozialer Angst und der Juckreizintensität sowie zur Erfassung der Kontrollvariablen (siehe unten) aus. Die Probanden erhalten für die Teilnahme an Studie 1 10 € und werden gebeten, ihre Kontaktdaten zu hinterlassen, um bei Interesse an Studie 2 und entsprechender Eignung kontaktiert werden zu können. Die Daten werden pseudonymisiert gespeichert (siehe unten).

Studie 2: Aus dem Pool der Teilnehmer an Studie 1 werden Patienten und hautgesunde Probanden entsprechend ihrer Ausprägung sozialer Angst ausgewählt (siehe Einschlusskriterien). Sie werden in einem Telefongespräch über Studie 2 und die Methode des Suction Blisters informiert. Zudem werden die Ein- und Ausschlusskriterien (siehe unten) per Telefon abgeklärt. Bei Einschluss in Studie 2 kommen 64 hautgesunde Probanden sowie 64 Psoriasis-Patienten an zwei Terminen in die Laborräume des Instituts für Med. Psychologie. Sie erhalten für die Teilnahme an Studie 2 eine Aufwandsentschädigung von 100 €. Am ersten Untersuchungstermin werden die Probanden an die Methode des Suction Blisters gewöhnt, indem sie ausführlich über das Vorgehen bei der Methode informiert werden, sich die Saugpumpe anschauen können und die Plexiglassaugkammer für 15 Minuten angelegt wird und Unterdruck appliziert wird. Dieser Termin dient lediglich der Habituation an diese Methode und nicht zur Erfassung der neuroinflammatorischen Parameter. Diese findet an einem zweiten Untersuchungstermin statt und dauert maximal 3.5 Stunden. Zum Ende des ersten Untersuchungstermins unterschreiben die Probanden die Einwilligungserklärung für Studie 2. Zu Beginn des zweiten Untersuchungstermins füllen sie zunächst Fragebogen aus (Erfassung der Kontrollvariablen, Erfassung von sozialer Angst und Juckreiz; siehe unten). Anschließend wird bei den Probanden die Methode des Suction-Blisters angewendet und somit Gewebe-Flüssigkeit gewonnen, um hierin die neuroinflammatorischen Parameter zu bestimmen (siehe ebenfalls unten).

# Welche Merkmale, Messgrößen (Variablen) werden beobachtet bzw. bestimmt?

*1) Soziale Angst*

Soziale Angst wird sowohl in Studie 1 als auch in Studie 2 mittels derselben Fragebögen erfasst. Es werden zwei Skalen zur Sozialen Angststörung (SOZAS; von Consbruch et al., 2016) eingesetzt. Bei ihnen handelt es sich um die Soziale-Interaktions-Angst-Skala (SIAS) und die Soziale Phobie-Skala (SPS). Diese beiden in der deutschen Sprache validierten Messinstrumente umfassen jeweils 20 Fragen, die auf einer 5-stufigen Skala beantwortet werden. Während die SIAS die Interaktionsangst erfasst, erfasst die SPS Angst in Bewertungssituationen. Die Items in beiden Fragebögen beziehen sich nicht auf einen bestimmten Zeitraum. In dieser Studie werden Probanden gemäß dem von Stangier et al., (1999) ermittelten Cut-Off Wert mit SIAS-Werten ≥ 26 als hoch sozial ängstlich (HSA) definiert. Personen, die in Studie 1 einen Prozentrang ≤ 25 aufweisen, gelten als niedrig sozial ängstlich (NSA).

*2) Juckreizintensität*

In Studie 1 werden nach der Erfassung der Kontrollvariablen und sozialen Angst sowohl die aktuelle Juckreizintensität als auch die Juckreizintensität innerhalb der letzten 24 Stunden vor dem Untersuchungstermin mittels visueller Analogskala (VAS 0-10) mit den beiden Polen „kein Juckreiz“ (0) und „schlimmster vorstellbarer Juckreiz“ (10) erfasst. Zudem wird bei den PS-Patienten nach dem Vorhandensein von chronischem Juckreiz gefragt und die aktuelle Juckreizintensität in Relation zur schlimmsten aufgrund von Psoriasis erlebten Juckreizintensität erhoben. Hierzu werden die Patienten gebeten, ihren aktuellen Juckreiz in Bezug zu setzen zu dem maximalen Juckreiz, den sie aufgrund von Psoriasis erlebt haben. Sie sollen angeben, ob der momentane Juckreiz genauso stark ist, minimal besser, etwas besser, deutlich besser oder sehr deutlich besser. In Studie 2 wird zu Beginn des zweiten Untersuchungstermins der aktuelle Juckreiz als Baseline-Wert, ebenfalls über die Verwendung der oben beschriebenen VAS ermittelt. Im Verlauf der Anwendung der Suction-Blister-Methode wird zudem im Abstand von 30 Minuten die Juckreizintensität per VAS (0-10) zu maximal sechs Messzeitpunkten bis zur Entstehung der Saugblasen und anschließenden Flüssigkeitsentnahme erfasst.

*3) Neuroinflammatorische Parameter*

Die Konzentration der neuroinflammatorischen Parameter IL-6, IL-17 und Substanz-P wird in der mittels Suction-Blister-Methode (siehe unten) gewonnenen Gewebeflüssigkeit mit Hilfe von ELISA-Assays (IBL International, Hamburg) entsprechend modifizierter Herstellerempfehlungen bestimmt. Ein umfassendes Panel von Mediatoren der angeborenen und erlernten Immunität könnte in zukünftigen, weiterführenden Untersuchungen mittels Cytometric Bead Array Analyse (Bender MedSystems, eBioscience, Frankfurt) bestimmt werden.

Die in Studie 2 eingesetzte Suction-Blister-Methode wurde vor mehr als 50 Jahren entwickelt (Kiistala, 1968) und ermöglicht eine schmerzfreie Ablation der oberen Schichten der Epidermis von der Dermis, wobei sich eine Blase oberhalb der Basalmembran bildet und es nicht zu einer Verletzung der darunterliegenden Strukturen kommt. Damit kommt es nicht zu Schmerzen, zu keiner Blutung oder sichtbaren anschließenden Heilung (z.B. Narbenbildung). Die Saugblasen werden durch einen konstanten Unterdruck von 200-300 mmHG erzeugt, mittels einer fein-einstellbaren Saugpumpe und einer für diesen Zweck speziell angefertigten Plexiglassaugkammer aus einer Werkstatt der Charité mit 3 kreisrunden, 8 mm durchmessenden Öffnungen entsprechend Abbildung 2. Dabei wird bis zum Entstehen der Blase über 1-3 Stunden Unterdruck appliziert. Bei diesem Vorgang kommt es zum Übertritt von interstitieller Flüssigkeit aus der Epidermis und Dermis in den Blasenhohlraum, inklusive der zwischenzellulär enthaltenen Proteine und sonstigen Botenstoffe wie Neurotransmittern, Neuropeptiden sowie Zytokinen (Niedzwiecki et al., 2018; Mias et al., 2018; Myles et al., 2018; Tobin et al., 2000). In der interstitiellen Flüssigkeit ist somit eine Vielzahl neuroinflammatorischer Parameter messbar. Die Flüssigkeit wird aus der Blase steril mittels Insulinspritze abgesaugt, in ein Eppendorfröhrchen transferiert, zentrifugiert und der Überstand bei -80°C bis zur Analyse der darin enthaltenen Botenstoffe gelagert. Für mögliche weiterführende molekularbiologische Analysen der mRNA Expression oder epigenetischen Methylierungsmustern werden die abzentrifugierten Zellen des Immunsystems sowie das Blasendach ebenfalls bei -80°C eingelagert. Dabei wird darauf verzichtet wie in anderen Studien explizit eine Immunreaktion vorab zu provozieren (Holm et al., 2018), sondern es werden die nativ vorhandenen Zellen unter Krankheits- und unterschiedlichen Belastungsbedingungen (hohe vs. niedrige soziale Angst) untersucht. Die Saugblase wird auf nicht-läsionaler Haut des Unterarms der Patienten erzeugt. Die Blase wird so induziert, dass sie sich im Abstand von mindestens 3 cm zur nächsten läsionalen Hautstelle befindet.


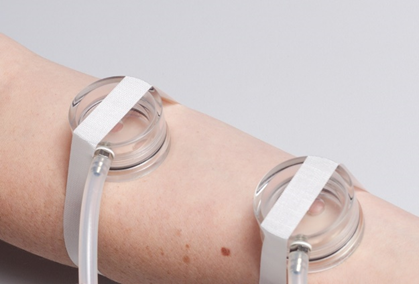

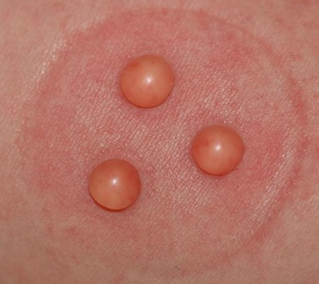


Abbildung 2. Anwendung der minimal-invasiven Suction-Blister-Methode, bei der durch Unterdruck Gewebeflüssigkeit in Blasen gesaugt wird und von dort schmerzfrei entnommen werden kann (Bildquelle: http://www.sit-skin.de/services/suction-blister-method)

**Was sind die zur Beantwortung der Fragestellung wesentlichen Variablen?**

- Soziale Angst (Studien 1 und 2)
- Juckreizintensität (Studien 1 und 2)
- Neuroinflammatorische Parameter IL-6, IL-17 und Substanz P (Studie 2)

**Mit Hilfe welcher Variablen sollen zusätzliche Informationen gewonnen werden**

Als Kontrollvariablen werden in Studie 1 und 2 personenbezogene Variablen (Alter, Geschlecht, BMI, Rauchen, Einnahme oraler Kontrazeptiva und Sozialstatus operationalisiert über den höchsten erreichten Schulabschluss) und der selbsteingeschätzte Schweregrad der Psoriasis während der letzten 24 Stunden vor Untersuchungsbeginn über den SA-PASI (Feldman et al., 1996) erhoben. Darüber hinaus wird in Studie 2 der aktuelle psychische Gesundheitszustand mittels der Kurzform des deutschen Gesundheitsfragebogens für Patienten (PHQ-D; Gräfe et al., 2004) sowie das generelle und momentane Angsterleben über das State-Trait-Angstinventar (STAI; Laux et al., 1981) erfasst. Auch werden Patienten, bei denen davon ausgegangen werden kann, dass die Ausschüttung neuroinflammatorischer Zytokine durch die Anwendung von Biologika verändert ist, von der Teilnahme an Studie 2 ausgeschlossen (siehe unten). Das Geschlecht wird innerhalb der Subgruppen „HSA“ und „NSA“ weitmöglich stratifiziert. Sollte es der Fall sein, dass das Geschlechterverhältnis in der HSA- und/oder NSA-Gruppe nicht ausgewogen ist, geht das Geschlecht als Kovariate in die Analysen ein. Zudem wird die Zeit, die es dauert, bis sich die Saugblase auf der Haut bildet, als Kontrollvariable erhoben.

**Welche Störgrößen können das Ergebnis beeinflussen**

Es sind keine Störgrößen bekannt, die das Ergebnis beeinflussen können, die nicht erfasst werden.

**Planung der biometrischen Auswertung und Überlegungen zum Stichprobenumfang**

Zur Beantwortung von Fragestellung 1 wird eine lineare hierarchische Regression durchgeführt, bei der im ersten Schritt die Kontrollvariablen und im zweiten Schritt die soziale Angst (gemessen über den SIAS und SPS) als potentielle Prädiktorvariablen eingehen. Kriteriumsvariable stellt die Juckreizintensität dar.

Zur Beantwortung von Fragestellung 2 wird eine Mediationsanalyse (Baron & Kenny, 1986; Preacher & Hayes, 2008) unter Einsatz des SPSS Makros „Process“ durchgeführt. In die Analyse werden Kontrollvariablen und soziale Angst als Prädiktorvariablen, neuroinfammatorische Parameter als Mediatoren und die Juckreizintensität als Kriteriumsvariable eingehen. Eine Berechnung der benötigten Stichprobengröße mittels G-Power (Faul et al., 2009) zur Beantwortung der zentralen Fragestellung 2 ergab, dass zur Durchführung der Mediationsanalyse 64 Patienten mit Psoriasis (n=32 mit niedriger und n=32 mit hoher sozialer Angst (SIAS-Wert ≥ 26) untersucht werden müssen, um kleine bis mittlere Effekte (f^2^=0.10) aufdecken zu können. Von kleinen bis mittleren Effekten ist auszugehen, da zum einen nur Extremgruppen untersucht werden und zum anderen in Vorgängerstudien kleine bis moderate Korrelationen zwischen Angst und Juckreizintensität bei Psoriasis-Patienten (z.B. Mrowietz et al., 2015) sowie zwischen neuroinflammatorischen Parametern und Juckreizintensität gefunden wurden (Konda et al., 2015). Um n=64 Patienten in Studie 2 einschließen zu können, werden 250 Psoriasis-Patienten im Rahmen von Studie 1 untersucht. Aus diesem Pool werden alle Patienten angesprochen, die hohe soziale Angstwerte (Wert im SIAS ≥ 26 (Cut-Off-Wert)) aufweisen und diejenigen, die niedrige soziale Angstwerte aufweisen (PR ≤ 25), und zur Teilnahme an Studie 2 motiviert. Die Ansprache der großen Zahl an Probanden ist notwendig, um n=64 Probanden in Studie 2 einschließen zu können.

Zur Beantwortung der Nebenfragestellungen werden einfaktorielle Kovarianzanalysen gerechnet.

**Falls eine Zwischenauswertung vorgesehen ist** Zwischenauswertungen sind nicht vorgesehen.

**Falls Design-Änderungen durchgeführt werden sollen/können** Designänderungen sind nicht vorgesehen.

**Ethik**

Probanden nehmen an dem geplanten Projekt auf freiwilliger Basis teil. Sie erhalten für die Teilnahme an Studie 1 10 € Probandengeld. Ihre Motivation besteht zudem darin, einen Beitrag zur Verbesserung des Kenntnisstandes zu Faktoren, die Juckreizintensität bei Psoriasis-Patienten mitbestimmen, zu leisten. Durch das Bearbeiten der Fragebögen sind sie keinerlei Risiken ausgesetzt. Auch die Anwendung der Suction-Blister-Methode ist nicht mit Risiken verbunden. Es handelt sich wie oben beschrieben um eine keine Schmerzen induzierende Methode, die keine Narbenbildung verursacht. Es ist lediglich möglich, dass durch die Methode ein leichter, transienter Juckreiz erzeugt wird, was mit erhoben wird.

**Datenschutz**

Die Bestimmungen des Datenschutzgesetzes werden streng befolgt. Die Eingabe der Fragebögen erfolgt in pseudonymisierter Form. Die Fragebögen sind also nur mit einem individuellen pseudonymisierten Code versehen und werden separat von den Einverständnisbögen aufbewahrt Es wird allerdings Zuordnungslisten geben, aus denen ein Rückschluss von den erhobenen Daten auf den Probanden möglich ist. Dies ist insofern notwendig, dass im Falle der Identifikation hoher oder niedriger sozialer Angst in der Fragebogenstudie (Studie 1) die Probanden erneut kontaktiert werden, um sie zur Teilnahme an Studie 2 zu motivieren. Nach dem Ausfüllen der Fragebögen werden diese separat von der Einverständniserklärung in im Institut für Medizinische Psychologie der Justus-Liebig-Universität Gießen in verschlossenen Schränken für eine Dauer von 10 Jahren aufbewahrt, da es sich um wissenschaftliche Primärdaten handelt. . Zur statistischen Auswertung werden die Daten in SPSS 26 (IBM Corp, 2016) eingegeben. Danach werden diese vernichtet. Die Einverständniserklärungen werden ebenfalls nach Einhaltung der zehnjährigen Aufbewahrungspflicht vernichtet. Die Probanden können ihr Einverständnis, an der Studie teilzunehmen, bis zur Abgabe der Fragebögen, ohne Angabe von Gründen widerrufen. Daraus entstehen ihnen keine Nachteile.

# Maßnahmen zur Gewährleistung der Patientensicherheit

Durch die Teilnahme an der Studie bestehen für die Probanden keinerlei psychische oder körperliche Gefahren.

**Qualitätsmanagement**

Die Fragebögen werden entsprechend der Richtlinien in den Testmanualen ausgewertet.

# Publikation

Die Veröffentlichung der Ergebnisse ist in Form einer Dissertation geplant. Zudem sollen die Ergebnisse in internationalen Journalen aus dem Bereich Psychosomatik und Dermatologie veröffentlicht werden.

**Soll das Studienprotokoll** **publiziert bzw. die Studie angemeldet** **werden**

Es ist vorgesehen, diese Studie beim Deutschen Register Klinischer Studien (DRKS) zu registrieren, da viele Journale für die Publikation die Angabe einer Clinical Trial Number fordern.

**Literatur**

1. Alpsoy E, Polat M, FettahlıoGlu-Karaman B, Karadag AS, Kartal-Durmazlar P, YalCın B, Emre S, Didar-Balcı D, Bilgic-Temel A, Arca E, Koca R, Gunduz K, Borlu M, Ergun T, Dogruk-Kacar S, Cordan-Yazici A, Dursun P, BilgiC O, Gunes-Bilgili S, Sendur N, Baysal O, Halil-Yavuz I, Yagcioglu G, Yilmaz E, Kavuzlu U, Senol Y. Internalized stigma in psoriasis: A multicenter study. J Dermatol. 2017;44:885-891.
2. Augustin M, Reich K, Glaeske G, Schaefer I, Radtke M. Co-morbidity and age-related prevalence of psoriasis: Analysis of health insurance data in Germany. Acta Derm Venereol. 2010;90:147-151.
3. Baron RM, Kenny DA. The moderator-mediator variable distinction in social psychological research: conceptual, strategic, and statistical considerations. J Pers Soc Psychol. 1986;51:1173-82.
4. Connor CJ, Liu V, Fiedorowicz JG. Exploring the physiological link between psoriasis and mood disorders. Dermatol Res Pract. 2015:409637. doi: 10.1155/2015/409637
5. Dalgard FJ, Svensson Å, Halvorsen JA, Gieler U, Schut C, Tomas-Aragones L, Lien L, Poot F, Jemec GBE, Misery L, Szabo C, Linder D, Sampogna F, Spillekom-van Koulil S, Balieva F, Szepietowski JC, Lvov A, Marron SE, Altunay IK, Finlay AY, Salek S, Kupfer J. Itch and mental health in dermatological patients across Europe: a cross sectional study in 13 countries. J Invest Dermatol. 2019; doi: 10.1016/j.jid.2019.05.034
6. Faul F, Erdfelder E, Buchner A, Lang AG. Statistical power analyses using G*Power 3.1: Tests for correlation and regression analyses. Behav Res Methods. 2009; 41:1149-1160.
7. Feldman SR, Fleischer AB, Reboussin DM, Rapp SR, Exum ML, Clark AR, Nurre L. The self-administered psoriasis area and severity index is valid and reliable. J Invest Dermatol. 1996;106:183-186.
8. Globe D, Bayliss MS, Harrison DJ. The impact of itch symptoms in psoriasis: results from physician interviews and patient focus groups. Health Qual Life Outcomes. 2009 Jul 6;7:62. doi: 10.1186/1477-7525-7-62
9. Gräfe K, Zipfel S, Herzog W, Löwe B. Screening psychischer Störungen mit dem “Gesundheitsfragebogen für Patienten (PHQ-D)” Diagnostica 2004; 50:171-181.
10. Holm LL, Vukmanovic-Stejic M, Blauenfeldt T, Benfield T, Andersen P, Akbar AN, Ruhwald M. A Suction Blister Protocol to Study Human T-cell Recall Responses In Vivo. J Vis Exp. 2018;138.e57554, doi:10.3791/57554
11. Kiistala U. Suction blister device for separation of viable epidermis from dermis. J Invest Dermatol. 1968; 50:129-137.
12. Konda D, Chandrashekar L, Rajappa M, Kattimani S, Thappa DM, Ananthanarayanan PH. Serotonin and interleukin-6: Association with pruritus severity, sleep quality and depression severity in Prurigo Nodularis. Asian J Psychiatr. 2015;17:24-28.
13. Lahousen T, Kupfer J, Gieler U, Hofer A, Linder MD, Schut C. Differences Between Psoriasis Patients and Skin-healthy Controls Concerning Appraisal of Touching, Shame and Disgust. Acta Derm Venereol. 2016;96:78-82.
14. Laux L, Glanzmann P, Schaffner P, Spielberger CD. STAI – Das State-Trait Angstinventar. 1981; Hogrefe: Göttingen.
15. Leff Gelman P, Mancilla-Herrera I, Flores-Ramos M, Saravia Takashima MF, Cruz Coronel FM, Cruz Fuentes C, Pérez Molina A, Hernández-Ruiz J, Silva-Aguilera FS, Farfan-Labonne B, Chinchilla-Ochoa D, Garza Morales S, Camacho-Arroyo I. The cytokine profile of women with severe anxiety and depression during pregnancy. BMC Psychiatry. 2019;19:104. doi: 10.1186/s12888-019-2087-6
16. Liang SE, Cohen JM, Ho RS. Psoriasis and suicidality: A review of the literature. Dermatol Ther. 2019;32:e12771. doi: 10.1111/dth.12771
17. Liu Y, Ho RC, Mak A. The role of interleukin (IL)-17 in anxiety and depression of patients with rheumatoid arthritis. Int J Rheum Dis. 2012;15:183-187.
18. Mias C, Le Digabel J, Filiol J, Gontier E, Gravier E, Villaret A, Nocera T, Questel E, Rossi AB, Redoulès D, Josse G. Visualization of dendritic cells' responses in atopic dermatitis: Preventing effect of emollient. Exp Dermatol. 2018;27:374-377.
19. Mrowietz U, Chouela EN, Mallbris L, Stefanidis D, Marino V, Pedersen R, Boggs RL. Pruritus and quality of life in moderate-to-severe plaque psoriasis: post hoc explorative analysis from the PRISTINE study. J Eur Acad Dermatol Venereol. 2015;29:1114-1120.
20. Muñoz M, Coveñas R. Involvement of substance P and the NK-1 receptor in human pathology. Amino Acids. 2014;46:1727-1250.
21. Myles IA, Anderson ED, Earland NJ, Zarember KA, Sastalla I, Williams KW, Gough P, Moore IN, Ganesan S, Fowler CJ, Laurence A, Garofalo M, Kuhns DB, Kieh MD, Saleem A, Welch PA, Darnell DA, Gallin JI, Freeman AF, Holland SM, Datta SK. TNF overproduction impairs epithelial staphylococcal response in hyper IgE syndrome. J Clin Invest. 2018;128:3595-3604.
22. Nattkemper LA, Tey HL, Valdes-Rodriguez R, Lee H, Mollanazar NK, Albornoz C, Sanders KM, Yosipovitch G. The Genetics of Chronic Itch: Gene Expression in the Skin of Patients with Atopic Dermatitis and Psoriasis with Severe Itch. J Invest Dermatol. 2018;138:1311-1317.
23. Niedzwiecki MM, Samant P, Walker DI, Tran V, Jones DP, Prausnitz MR, Miller GW. Human Suction Blister Fluid Composition Determined Using High-Resolution Metabolomics. Anal Chem. 2018;90:3786-3792
24. Preacher KJ, Hayes AF. Asymptotic and resampling strategies for assessing and comparing indirect effects in multiple mediator models. Behav Res Methods. 2008;40:879-891.
25. Sanders KM, Akiyama T. The vicious cycle of itch and anxiety. Neurosci Biobehav Rev, 2018; 87:17-26.
26. Sanders KM, Sakai K, Henry TD, Hashimoto T, Akiyama, T. A Subpopulation of Amygdala Neurons Mediates the Affective Component of Itch. J Neurosci. 2019;39:3345-3356.
27. Schut C. Stressbewältigung bei Neurodermitis: Psychophysiologische Effekte. 2013. Dissertation. http://geb.uni-giessen.de/geb/volltexte/2013/9914/
28. Schut C, Bosbach S, Gieler U, Kupfer, J. Personality traits, depression and itch in patients with atopic dermatitis in an experimental setting: a regression analysis. Acta Derm Venereol. 2014a; 94: 20-25.
29. Schut C, Felsch A, Zick C, Hinsch KD, Gieler U, Kupfer J. Role of illness representations and coping in patients with atopic dermatitis: a cross-sectional study. J Eur Acad Dermatol Venereol. 2014b;28:1566-1571
30. Schut C, Muhl S, Reinisch K, Claßen A, Jäger R, Gieler U, Kupfer J. Agreeableness and self-consciousness as predictors of induced scratching and itch in patients with psoriasis. Int J Behav Med. 2015a;22:726-734
31. Schut C, Weik U, Tews N, Gieler U, Deinzer R, Kupfer J. Coping as mediator of the relationship between stress and itch in patients with atopic dermatitis: a regression and mediation analysis. Exp Dermatol. 2015b;24:148-150.
32. Schut C, Rädel A, Frey L, Gieler U, Kupfer, J. Role of personality and expectations for itch and scratching induced by audiovisual itch stimuli. Eur J Pain. 2016;20:14-18.
33. Schut C, Reinisch K, Classen A, Andres S, Gieler U, Kupfer, J. Agreeableness as Predictor of Induced Scratching in Patients with Atopic Dermatitis: A Replication Study. Acta Derm Venereol. 2018; 98:32-37.
34. Schut C, Dalgard FJ, Halvorsen JA, Gieler U, Lien L, Aragones LT, Poot F, Jemec GBE, Misery L, Kemény L, Sampogna F, van Middendorp H, Balieva F, Linder D, Szepietowski JC, Lvov A, Marron SE, Altunay IK, Finlay AY, Salek S, Kupfer J. Occurrence, Chronicity and Intensity of Itch in a Clinical Consecutive Sample of Patients with Skin Diseases: A Multi-centre Study in 13 European Countries. Acta Derm Venereol. 2019; 99:146-151.
35. Sommer R, Augustin M, Mrowietz U, Topp J, Schäfer I, von Spreckelsen R. Perception of stigmatization in people with psoriasis-qualitative analysis from the perspective of patients, relatives and healthcare professionals. Hautarzt. 2019;70:520-526.
36. Stangier U, Heidenreich T, Berardi A, Golbs U, Hoyer J. Die Erfassung sozialer Phobie durch die Social Interaction Anxiety Scale (SIAS) und die Social Phobia Scale (SPS). Zeitschrift für Klinische Psychologie 1999;28:28-36
37. Szepietowski JC, Reich A. Pruritus in psoriasis: An update. Eur J Pain. 2016;20:41-6.
38. Théréné C, Brenaut E, Barnetche T, Misery L. Efficacy of Systemic Treatments of Psoriasis on Pruritus: A Systemic Literature Review and Meta-Analysis. J Invest Dermatol. 2018;138:38-45.
39. Tobin DJ, Swanson NN, Pittelkow MR, Peters EM, Schallreuter KU. Melanocytes are not absent in lesional skin of long duration vitiligo. J Pathol. 2000;191:407-416.
40. von Consbruch K, Stangier U, Heidenreich T. SOZAS – Skalen zur Sozialen Angststörung. 2016; Hogrefe: Göttingen.
41. Wang X-D, Yang G, Bai Y, Feng Y-P, Li H. The behavioral study on the interactive aggravation between pruritus and depression. Brain Behav. 2018;8:e00964. doi: 10.1002/brb3.964
42. Zachariae R, Lei U, Haedersdal M, Zachariae C. Itch severity and quality of life in patients with pruritus: preliminary validity of a Danish adaptation of the itch severity scale. Acta Derm Venereol. 2012;92:508-14.
43. Zeidler C, Pereira MP, Huet F, Misery L, Steinbrink K, Ständer S. Pruritus in Autoimmune and Inflammatory Dermatoses. Front Immunol. 2019;10:1303. doi: 10.3389/fimmu.2019.01303
44. Zhao X, Yu C, Ye F, Wang YG, Mei QY, Ma Q, Cui WG, Zhou WH. Chronic itch impairs mood and HPA axis function in mice: modulation by CRFR1 antagonist. Pain 2018;159: 2201-2213
